# London dispersion dominating diamantane packing in helium nanodroplets†

**DOI:** 10.1039/d1cp03380h

**Published:** 2021-09-20

**Authors:** Jasna Alić, Roman Messner, Florian Lackner, Wolfgang E. Ernst, Marina Šekutor

**Affiliations:** Department of Organic Chemistry and Biochemistry, Ruđer Bošković Institute Bijenička cesta 54 10 000 Zagreb Croatia msekutor@irb.hr; Institute of Experimental Physics, Graz University of Technology Petersgasse 16 8010 Graz Austria florian.lackner@tugraz.at wolfgang.ernst@tugraz.at

## Abstract

Diamantane clusters formed inside superfluid helium nanodroplets were analyzed by time-of-flight mass spectrometry. Distinct cluster sizes were identified as “magic numbers” and the corresponding feasible structures for clusters consisting of up to 19 diamantane molecules were derived from meta-dynamics simulations and subsequent DFT computations. The obtained interaction energies were attributed to London dispersion attraction. Our findings demonstrate that diamantane units readily form assemblies even at low pressures and near-zero Kelvin temperatures, confirming the importance of the intermolecular dispersion effect for condensation of matter.

## Abstract 2

Diamantane molecules deposited into helium nanodroplets form magic number clusters and the process is driven by dispersion interactions.

## Graphical abstract

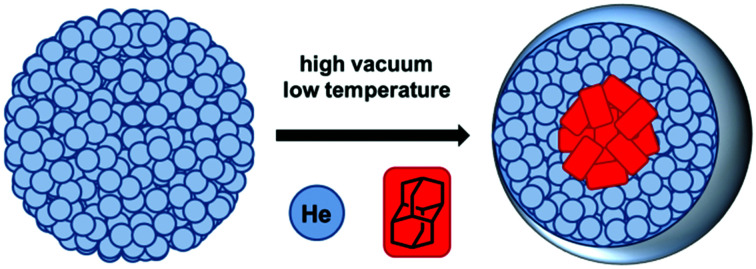

## Introduction

Superfluid helium nanodroplets (HNDs) are unique hosts for the study of weak interactions between molecules. The cold and sub-micron sized helium aggregates are produced by expanding gaseous helium into high vacuum at cryogenic temperatures^1–3^ and can be used to trap atoms and molecules which they pick up upon collision. HNDs are also utilized as small reaction chambers where large clusters can form, with the released binding energy being dissipated by the evaporation of helium atoms.^4–9^ Since helium has very low polarizability that results in weak He⋯He interactions, it becomes superfluid at low pressures and near-zero Kelvin temperatures, with emerging properties like vanishing viscosity and high heat conductivity.^10^ HNDs, therefore, have an almost negligible perturbative effect on dopant molecules and are an ideal medium for trapping weakly binding van der Waals complexes.^11,12^ In previous experiments we already used the exceptional properties of HNDs to investigate molecular clusters like (V_2_O_5_)_*n*_^8^ or the very weakly bound alkali triplet-dimers^13^ and quartet-trimers.^14^ Particles consisting of many units of doped molecules immersed inside HNDs are usually well-defined and can be deposited in a soft manner on surfaces for detection, thereby offering a non-destructive way to analyze complexes held together by quite weak forces.

Due to their properties, HNDs pose a promising matrix to create self-organized clusters of diamondoid molecules. Diamondoids are nanometer-sized hydrocarbons that are readily found in nature^15^ and have unique properties due to their structural similarity to the diamond crystal lattice.^16^ In contrast to diamond, diamondoids are hydrogen-terminated saturated organic molecules that can be selectively functionalized and applied in nanomaterial design.^17^ Since diamondoids are rather bulky and rich in C–H bonds, they readily engage in London dispersion (LD)^18^ intermolecular interactions with each other. However, LD is an inherently weak interaction and many LD contacts between molecules are needed to produce an observable macroscopic effect. What is more, solvent molecules often disrupt LD interactions and make the experimental LD quantification^19^ even more difficult.^20^ Since HNDs are a unique non-disruptive system that enables basically undisturbed cluster formation of added molecules, we envision them as a means to analyze and elucidate the structure of LD complexes of diamantane.

We previously studied the effect of LD interactions on diamondoid self-assembly on metal surfaces using scanning tunneling microscopy (STM) and atomic force microscopy (AFM) in combination with computational tools.^21–23^ We indeed found that LD interactions were responsible for on-surface organization and cluster formation of such bulky molecules. However, the nature of the experiment limited our study to only two dimensions since diamondoid molecules needed to be deposited on planar surfaces for single molecule detection. Our interest in LD interactions between diamondoids in a 3D environment was partially inspired by recent work of Scheier and coworkers, that offered insight into the aggregation behavior of the smallest diamondoid adamantane in helium nanodroplets.^24,25^ Furthermore, the behavior and cluster formation of diamondoids in a 3D environment at extremely low temperatures and pressures is also of interest from the perspective of astrochemistry since recent reports confirmed the presence of diamondoid molecules in space and the HND environment can mimic such interstellar conditions.^26,27^ Examples of other hydrocarbons and some other small molecules studied in HNDs exist,^3^*e.g.*, methane,^28,29^ ethane,^30^ haloalkanes,^31,32^ ethylene,^33^ benzene,^34^ fullerene,^35–41^ alcohols and ethers,^42^ methanol,^43^ triphenylmethanol,^44^ formamide,^45^*etc.* However, none of these molecules are as bulky as diamondoid compounds and consequently cannot engage in numerous intermolecular C–H bond contacts that facilitate a strong LD effect. In the scope of this study we therefore explored the cluster formation of diamantane in HND conditions. The obtained experimental results were strongly supported by our computational analysis, with special emphasis on the observed cluster sizes with large abundances, *i.e.*, the magic numbers up to 19, which is challenging for large assemblies consisting of many C_14_H_20_ molecules. We are confident that our computational approach adequately accounts for the observed diamantane cluster stability and can be more broadly applied for conglomeration prediction of similar molecular systems.

## Experimental

### Procedures and methods

#### Helium nanodroplets

**Fig. 1:**
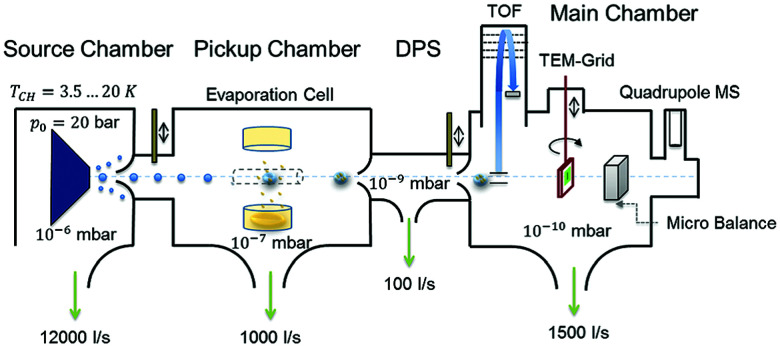
Schematic representation of the experimental setup used for HNDs generation and diamantane cluster synthesis. Reproduced from ref. 8 – published by The Royal Society of Chemistry.

**Fig. 2:**
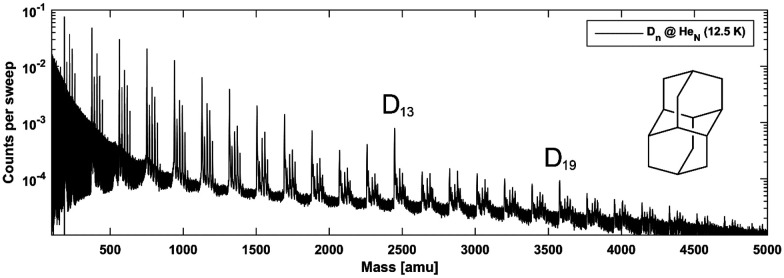
A recorded mass spectrum of He droplets doped with diamantane. Clusters with *n* = 13 and *n* = 19 units of diamantane show a higher abundance, suggesting increased stability. *E*_el_ = 90 eV, *I*_em_ = 6.8 μA, *T*_He_ = 12.5 K, *p*_He_ = 60 bar.

The apparatus used for the generation of the HNDs is described in detail in ref. 8, 9 and depicted in Fig. 1. In short, pressurized high purity He (99.9999%) is cooled to temperatures below 20 K by a closed-cycle refrigerator (Sumitomo RDK-408D2) and expanded through a 5 μm nozzle into high vacuum. During this process the gaseous He condenses into small superfluid droplets. At the expansion conditions used in the experiments (*p*_He_ = 60 bars, *T*_He_ = 11.5–12.5 K) He droplets with a mean diameter of 40 to 60 nm, consisting of about 1 × 10^6^ to 3 × 10^6^ He atoms, are formed.^1,3^ Subsequently, the beam passes a skimmer and the helium droplets pick up the desired dopant species in a separately pumped chamber. Here, we dope the droplets with diamantane (>98.0%, Tokyo Chemical Industry Co., Ltd) using a heated gas-pickup cell (100 °C), which is connected to a heated reservoir (70 °C) *via* a precision leak valve. The doped He droplets then enter the differentially pumped analysis chamber, where a reflectron time-of-flight mass spectrometer KAESDORF RTF50 is utilized to record mass spectra. Upon electron impact ionization diamantane cluster ions are expelled from the He droplets and can be detected at the corresponding mass channel. The employed emission current (*I*_em_) and ionization energy (*E*_el_) are *I*_em_ = 6.8 μA and *E*_el_ = 90 eV, respectively. Fig. 2 depicts a recorded mass spectrum with He intensities displayed on a logarithmic scale. Clusters with *n* = 13 and *n* = 19 show a higher abundance. Helium droplets were produced at nozzle conditions of *p*_He_ = 60 bar and *T*_nozz._ = 12.5 K.

#### Theoretical methods

The semi-empirical quantum mechanical GFN2-xTB method^46,47^ was used for constrained meta-dynamics (MTD) simulation^48^ of diamantane clusters lasting for 100 ps with a timestep of 1 fs at a temperature of 0.4 K. The constraint was achieved by using a repulsive potential to avoid cluster dissociation while allowing for freedom of movement of the diamantane cages inside the cluster sphere. Geometry optimizations of diamantane hydrocarbon (D), diamantyl carbocation (Dp) and the corresponding dimer structure (CL2), starting from the minima obtained by GFN2-xTB, were performed with the Orca 4.2.1 program package^49,50^ using the B3LYP-D3(BJ)/def2-TZVPP level of theory,^51,52^ and the obtained minima were verified by frequency computations. Single point computations on the same level of theory for CL13 and CL19 were done on the geometries from the GFN2-xTB optimization at 0.4 K. Additional single point computations were performed using the HF-3c,^53^ PBEh-3c,^54^ and ωB97X-gCP-D3(BJ)/def2-TZVPP^55^ levels of theory. Note that D3(BJ) dispersion correction,^51,52^ three-body dispersion contributions term implemented in Orca as well as geometrical counterpoise (gCP) correction^56^ were employed to account for subtle intermolecular interactions and mitigate the basis-set superposition errors, respectively. Lastly, highly accurate single-point interaction energy for CL2 was computed using the *ab initio* TightPNO-DLPNO-CCSD(T)/cc-pVTZ^57–60^ level of theory. NCI plots were obtained using Multiwfn 3.6^61^ and visualized by VMD software.^62^

## Results and discussion

In their study of adamantane, Scheier and coworkers found certain irregularities within the cluster abundances in their time-of-flight mass spectra, which they interpreted as magic numbers, belonging to particularly stable geometries.^24^ Since these reported specific cluster sizes appeared to be independent of the overall cluster charge, we rationalize that these experimental observations strongly point towards the influence of LD interactions on adamantane packing. For the singly charged cationic clusters the observed magic numbers were 13, 19, 38, 52, *etc.* It was proposed that the first magic number 13 occurs because an icosahedron structure spontaneously forms when attractive interactions act between particles; entropy and spherical confinement usually suffice for icosahedron formation.^63^ In the case of adamantane, the packing around the adamantyl cation leads to the first icosahedral shell consisting of neutral adamantane molecules (*n* = 13) and later to the formation of a nested icosahedron (*n* = 19). This more or less intuitive explanation should however be confirmed by quantitative computations and molecular modelling.

Mass spectra recorded for diamantane clusters embedded in helium droplets (see Fig. 2) suggest a very similar behavior since for this species the cluster sizes *n* = 13 and *n* = 19 also show increased abundancies. The finding of identical magic numbers is quite telling, considering the size difference and somewhat lower symmetry of diamantane (*D*_3d_) compared to adamantane (*T*_d_), and points towards intermolecular dispersion attraction as a main governing factor for binding and complex formation in both cases. Note that each diamantane cluster peak is accompanied by several additional peaks as a consequence of residual water pickup at the background pressure of 10^−7^ mbar in the pick-up chamber.^8^

**Fig. 3:**
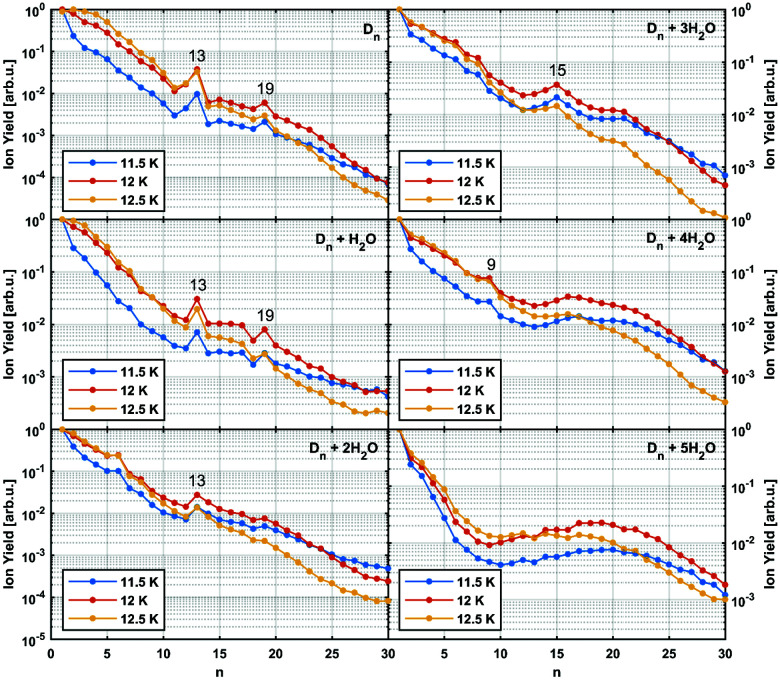
Normalized abundance of clusters consisting of n units of diamantane and additional water molecules. Particularly stable structures are marked with their cluster size. *E*_el_ = 90 eV, *I*_em_ = 6.8 μA, *p*_He_ = 60 bar.

In Fig. 3 the intensity of individual mass peaks that correspond to the pure diamantane cluster and diamantane clusters with several H_2_O molecules (18 amu) attached are plotted as a function of the cluster size *n* = 1–30. For three different He droplet sizes, adjusted by setting the nozzle temperature to 11.5 K, 12 K and 12.5 K, (corresponding to approximately 3 × 10^6^, 2 × 10^6^ and 1 × 10^6^ He atoms per droplet, respectively) mass spectra were captured, however, with very little differences. As discussed above, pure diamantane clusters with *n* = 13 and *n* = 19 show higher abundancies, just like it was the case for adamantane clusters. With an additional H_2_O molecule a similar trace is observed, exhibiting the same magic numbers. With the addition of more and more water molecules the magic numbers soften and other stable structures emerge. For example, **D**_*n*_ + 3H_2_O has a local maximum at *n* = 15 and for **D**_*n*_ + 4H_2_O a weak peak at *n* = 9 can be observed. **D**_*n*_ + 5H_2_O is the complex with the highest number of attached water molecules that could be analyzed. However, in this case a clear magic number can no longer be identified, except for a broad feature around *n* = 19. This is expected since the increase in water molecules effectively breaks up the existing non-polar network and replaces it with much stronger hydrogen bonding interactions acting between the introduced water molecules. Surprisingly, we do not see any doubly charged clusters **D**_*n*_^2+^ up to the investigated size of *n* = 60. These species are very pronounced for adamantane clusters with more than 19 units.^24^

It was observed that smaller adamantane clusters can also uptake water molecules inside their aggregates, which leads to a subsequent erosion of the parent hydrocarbon cluster.^25^ As already mentioned, this experimental observation is expected since water molecules form their own clusters *via* hydrogen bonds and therefore destroy a much loosely bound LD network that initially hosted them. Note, however, that hydration of the adamantane cluster (*n* = 13) first results in the replacement of an adamantane moiety with a water molecule conglomerate, (H_2_O)_21_, but manages to preserve the initial structural integrity of the system. What was not observed, however, was full emersion of a single adamantane molecule into a structured water network and this finding is also expected since adamantane is a highly lipophilic compound. We noticed a similar behavior in case of diamantane clusters. Even though it was not possible to resolve the addition of 21 water molecules with our TOF spectrometer, a clear trend away from the magic numbers of pure diamantane clusters can be seen (see Fig. 3) when small amounts of water are added. With three added water molecules the **CL13** and **CL19** show no anomalous abundance anymore; instead the cluster **D**_15_ + 3H_2_O shows enhanced stability. This indicates that stronger hydrogen bond interactions indeed start to dictate the overall structure of the cluster.

To gain more structural insight into the packing of diamantane molecules in HNDs, we performed a computational analysis of the three smallest experimentally found magic number clusters, **CL2**, **CL13** and **CL19**. The computed clusters consist of a diamantyl cation with a positive charge in the tertiary medial position surrounded by a corresponding number of neutral diamantane hydrocarbons. We chose the medial diamantyl cation (position 1 of the diamantane cage) for our computational study since it was shown that this cation was more stabilized by charge delocalization and therefore was lower in energy when compared to its apical counterpart (position 4 of the diamantane cage).^64^ Since the clusters of interest are somewhat large systems with many degrees of freedom, we first applied a semi-empirical tight-binding based quantum mechanical GFN2-xTB method^46,47^ to obtain preliminary geometries through a constrained meta-dynamics (MTD) simulation^48^ of clusters at 0.4 K (obtained trajectories depicted on Fig. S1–S3, ESI†). After getting the starting structures we proceeded with DFT computations and the results are shown in Table 1, with more details in the ESI.† For the optimization (B3LYP-D3(BJ)/def2-TZVPP level of theory) as well as single point computations we applied Grimme's D3 correction for dispersion interactions^51^ with Becke Johnson (BJ) damping^52^ and geometrical counterpoise (gCP) correction^56^ to mitigate the basis-set superposition error (BSSE). We also tested HF-3c, a fast Hartree–Fock based method, and PBEh-3c that were both developed for computation of interaction energies of non-covalent complexes and that inherently include three correction terms: for London dispersion interactions, for the BSSE and a short-ranged correction term to deal with basis set deficiencies which occur when using small or minimal basis sets. Thus, for our cluster interaction energy screening, we chose functionals based on our previous experience^21,22^ and on recent studies of DFT functional reliability.^65^ Lastly, we employed a highly accurate but more time-consuming DLPNO-CCSD(T) *ab initio* method using tight PNO settings recommended for weak complexes in conjunction with a cc-pVTZ basis set in order to obtain a more precise value for interaction energy of **CL2**.

**Table 1:** Interaction energies, Δ*H*(0 K), of diamantane clusters in kcal mol^−1 ^a^,^^b^

Level of theory	**CL2**	**CL13**	**CL19**
GFN2-xTB	−6.0	−72.6	−114.4
HF-3c	−7.6	−94.6	−149.4
PBEh-3c	−8.1	−102.0	−152.8
B3LYP-gCP-D3(BJ)/def2-TZVPP	−8.4	−96.8	−149.5
B3LYP-gCP-D3(BJ)-ABC/def2-TZVPP	−8.0	−87.7	−135.4
ωB97X-gCP-D3(BJ)/def2-TZVPP	−6.6	−93.3	−144.5
TightPNO-DLPNO-CCSD(T)/cc-pVTZ	−7.5	n.d.	n.d.

a Interaction energies are defined as a difference between the energy of the cluster and the energy of the corresponding number of diamantane moieties.

b ZPVE taken from GFN2-xTB computations.

The obtained interaction energies for diamantane clusters are of comparable values for all levels of theory we used (Table 1). For example, a stabilization of −7.5 kcal mol^−1^ for **CL2** at the TightPNO-DLPNO-CCSD(T)/cc-pVTZ level of theory is in good agreement with the obtained DFT energies and even comparable to a somewhat preliminary GFN2-xTB method. Inclusion of dispersion correction in our DFT computations was of course crucial due to the nature of the system under study and its necessity was demonstrated by performing a proof-of-principle optimization using the dispersion uncorrected B3LYP-gCP/def2-TZVPP level of theory (Table S2, ESI†). As expected, we obtained the energetic stabilization amounting to only −0.8 kcal mol^−1^ for **CL2** accompanied by geometrical perturbation, *i.e.*, distancing of the two diamantane cages in the cluster (Table S6, ESI†), which indeed justifies our chosen dispersion corrected levels of theory as well as confirms our hypothesis of LD being the main driving force for the interaction. Note that we also validated our DFT approach by testing the applied geometrical counterpoise (gCP) correction.^56^ Since our computations involved quite large systems, *e.g.*, **CL19** consists of 645 atoms, and can be a challenge for larger basis sets, we critically evaluated our medium sized basis sets to see whether the BSSE has a decisive effect on the obtained energy values. Upon using the B3LYP and ωB97X functionals with a wide range of basis sets and corrected for both the dispersion and gCP effects (Table S3, ESI†), we found that the differences in the interaction energies with and without gCP correction are equal or smaller than the differences resulting from using different levels of theory (smaller *vs.* medium sized basis sets). Despite that, the use of gCP correction increases the accuracy of our results obtained by using medium sized basis sets and was therefore consistently applied (Table 1). Consequently, we can claim that the favorable interaction energies for the computed diamantane clusters are indeed a result of intermolecular LD stabilization and are not an overbinding artefact of the applied computational method. Lastly, we also tested the influence of the three-body dispersion contributions term on the values of interaction energies and found that the obtained energies are again comparable. For example, computed interaction energies using the B3LYP-gCP-D3(BJ)/def2-TZVPP level of theory with and without the three-body dispersion contributions term for **CL2** amount to −8.0 and −8.4 kcal mol^−1^, respectively (Table 1). The differences in those values are thus similar to the ones afforded by using different levels of theory of comparable accuracy.

**Fig. 4:**
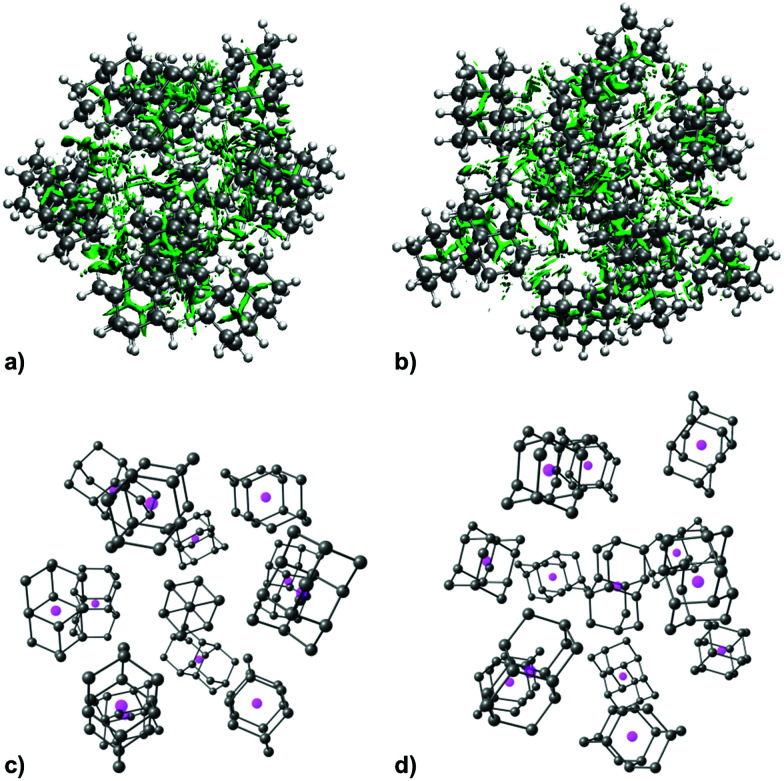
NCI plots of the computed structure of **CL13** with non-covalent interactions depicted in green, (a) top view, (b) side view, and the corresponding diamantane molecules depicted without hydrogen atoms for clarity with their geometrical centers represented with magenta spheres.

**Fig. 5:**
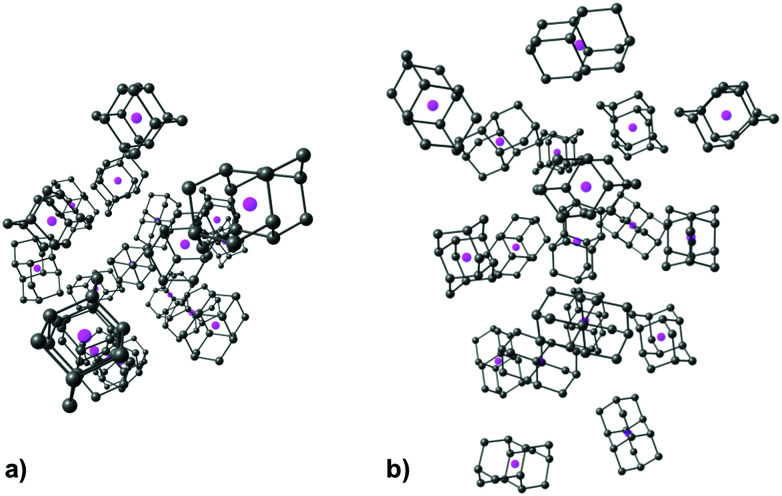
Computed structure of **CL19** depicted for clarity without diamantane hydrogen atoms and with cage geometrical centers represented with magenta spheres, (a) top view, (b) side view.

As expected, increase in cluster size also leads to the rise of interaction energies as LD effects gain strength upon multiplication of intermolecular close contacts. These areas of dense close contacts between diamantane molecules are depicted on Fig. S4 (ESI†) that visualizes non-covalent interactions (NCIs) obtained from DFT computations. The computed stable structure of **CL13** consists of a central 1-diamantyl cation surrounded by six diamantanes in the medial plane of the central cage molecule and by three diamantanes both on the top and on the bottom of the apical diamantane cage positions (Fig. 4). Although this structure is not exactly an icosahedron, it nevertheless utilizes the space around the central diamantyl cation very efficiently. It appears that the cluster's spatial arrangement is a consequence of the LD driving force that tries to maximize the number of close contacts between the C–H bonds and reduces the emptiness of the surroundings as much as possible. Similar can be said for **CL19** that also tries to engage in as many contacts between the diamantane moieties as available but despite that adopts a more elongated and, it appears, less ordered shape (Fig. 5). Such decrease in the cluster's structural order makes for more flexible conglomerates, which is also in line with our experimental findings where the relative abundance of **CL13** compared to its neighbors is much higher than that of **CL19**. This observation demonstrates that upon the increase of condensing diamantane subunits the cages readily arrange in similar clusters whose size and orientation heavily depend on the favorable LD interactions that the diamantane molecules engage in.

Based on the presented experimental findings that are additionally supported with molecular modelling results, we can confidently accredit the formation of diamantane clusters in HND conditions to beneficial LD intermolecular interactions acting between bulky diamantane molecules. However, the question remains why the experimentally observed magic numbers are special in terms of their abundance. A simplified rigid sphere model of atom packing cannot account for many-body effects and does not fully predict all magic numbers even for atomic HND clusters, let alone for molecular HND conglomerates like the case is here. Still, one possible explanation for this phenomenon could be the influence of inherent helium atom packing on the initial cluster formation. Namely, HNDs are reminiscent of typical condensed phase in some properties, most notable being the occurrence of somewhat ordered atom grouping,^12^ meaning that the initial arrangement of He atoms may influence the conglomeration process of diamantane molecules as they are gradually being deposited in the emerging clusters. In other words, pre-existing condensed phase arrangement of spherical helium atoms would govern the cluster growth towards the observed magic number conglomerates which would therefore be in higher abundance. While only a speculation at this point, it would be a plausible explanation for the observed occurrence of magic number clusters both for adamantane and diamantane molecules. Note, however, that gradual exclusion of the surrounding He atoms from cluster structures during their growth is still a consequence of better supramolecular stabilization enabled by beneficial intermolecular LD interaction acting between hydrocarbon cages. To illustrate, as the first diamantane is deposited in the nanodroplet, it becomes surrounded with a shell of He atoms which engage in LD interactions with the molecule. As more heliophilic diamantanes are further incorporated into the insides of the nanodroplet, they engage in mutual interaction since bulky hydrocarbons are typically good dispersion energy donors and outdo light helium atoms. Such energetic stabilization also explains the persistence of the clusters even upon their subsequent ionization and detection in the instrument chamber when all helium atoms are removed.

## Conclusions

We used superfluid helium nanodroplets as an ideal medium to explore clusters consisting of diamantane molecules by means of mass spectrometry. Since diamondoids are in principle good dispersion energy donors and readily engage in intermolecular LD interactions, they could successfully overcome weaker LD binding with helium atoms present in between dopant molecules, as evidenced by spontaneous cluster conglomeration. Additionally, magic number clusters were successfully identified and characterized. The experimental findings were supported by MTD and DFT computations, providing feasible cluster structures. Our quantum mechanical modelling approach successfully accounted for the structures of the aggregates despite their large size in terms of atom numbers, an accomplishment not so common in the exploration of doped helium droplets where the focus has up to now mostly been on individual atoms and small organic molecules. We also quantitatively demonstrated that dispersion interactions indeed dominate molecule packing in these clusters as we evaluated the corresponding interaction energies. Based on our results, we can with reasonable confidence extrapolate that bulky hydrocarbon molecules like diamantane readily form conglomerates even at HND conditions, illustrating the power of inherently weak forces in aggregation processes leading to bulk matter.

## Author contributions

The manuscript was written through contributions of all authors. All authors have given approval to the final version of the manuscript.

## Conflicts of interest

There are no conflicts to declare.

## Supplementary Material

CP-023-D1CP03380H-s001

CP-023-D1CP03380H-s002
