# Supplementary material for: London dispersion dominating diamantane packing in helium nanodroplets
Source: Phys Chem Chem Phys. 2021 Sep 20;23(38):21833–9. doi: 10.1039/d1cp03380h (PMC8494270; doi:10.1039/d1cp03380h)
Supplement: CP-023-D1CP03380H-s002 [file CP-023-D1CP03380H-s002.pdf]

Electronic Supplementary Material (ESI) for Physical Chemistry Chemical Physics.
This journal is © the Owner Societies 2021

Supporting information

London Dispersion Dominating Diamantane Packing in Helium Nanodroplets

Jasna Alić,[a, ‡] Roman Messner,[b, ‡] Florian Lackner,[b,*] Wolfgang E. Ernst,[b,*] Marina Šekutor,[a,*]

[a] Department of Organic Chemistry and Biochemistry, Ruđer Bošković Institute, Bijenička cesta 54, 10 000 Zagreb, Croatia, msekutor@irb.hr

[b] Institute of Experimental Physics, Graz University of Technology, Petersgasse 16, 8010 Graz, Austria, wolfgang.ernst@tugraz.at, florian.lackner@tugraz.at

‡Both contributors are considered first authors.

1. Computations

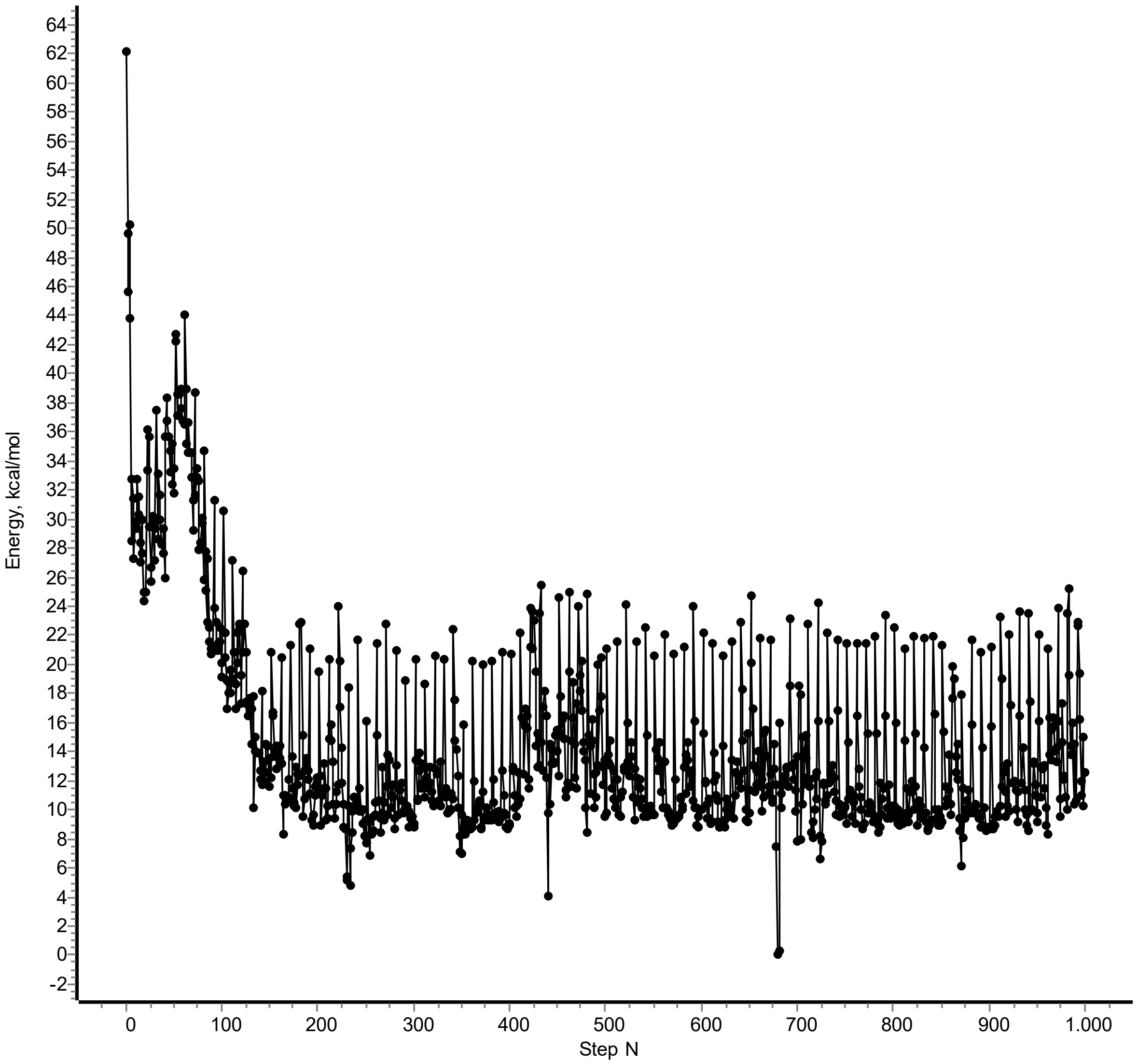

Figure S1. Trajectory from a constrained meta-dynamics (MTD) simulation of **CL2** using the GFN2-xTB method lasting for 100 ps with a timestep of 1 fs at a temperature of 0.4 K.

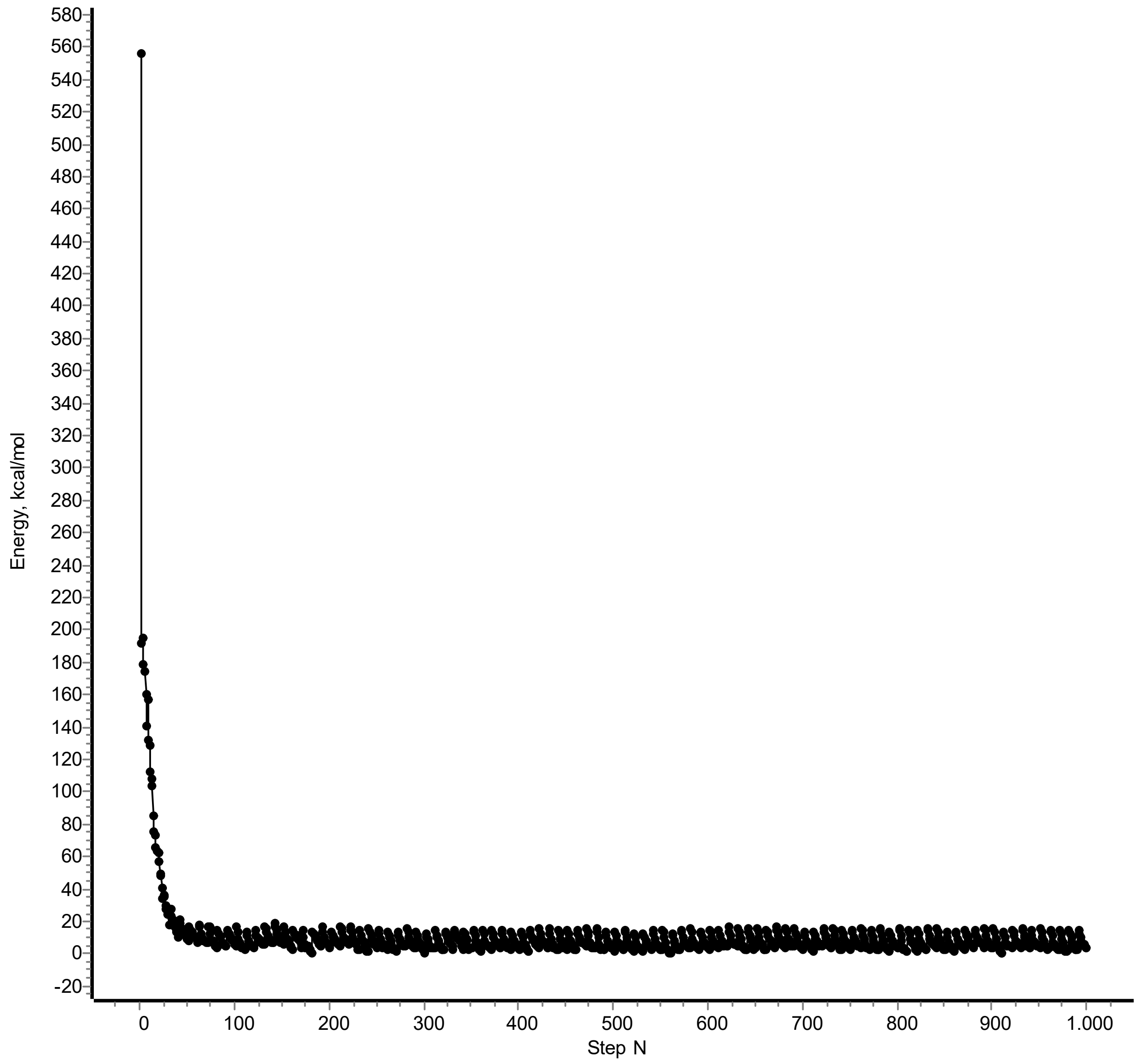

Figure S2. Trajectory from a constrained meta-dynamics (MTD) simulation of **CL13** using the GFN2-xTB method lasting for 100 ps with a timestep of 1 fs at 0.4 K.

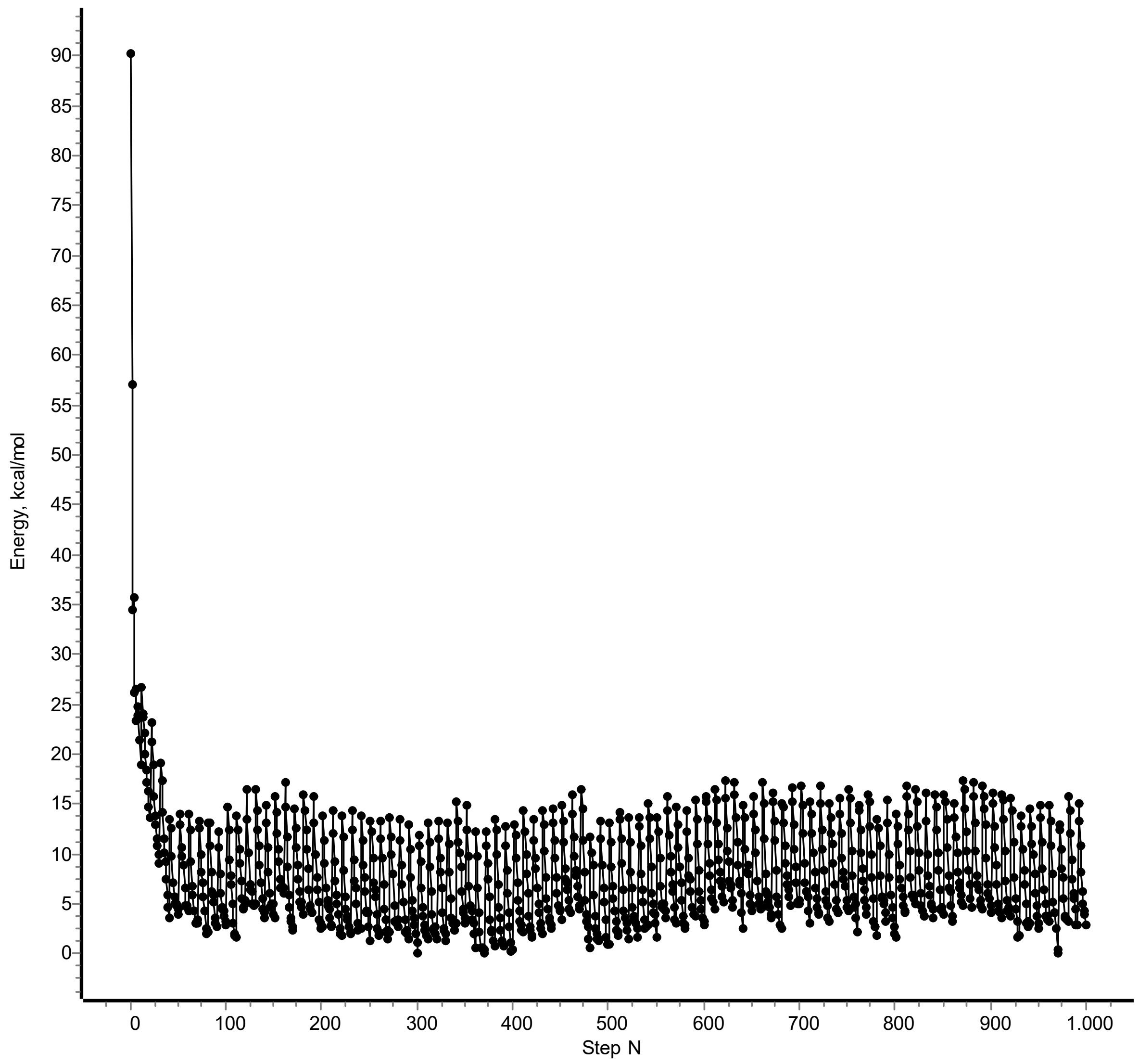

Figure S3. Trajectory from a constrained meta-dynamics (MTD) simulation of **CL19** using the GFN2-xTB method lasting for 100 ps with a timestep of 1 fs at 0.4 K.

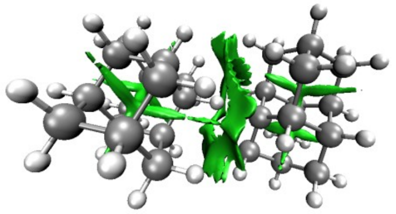

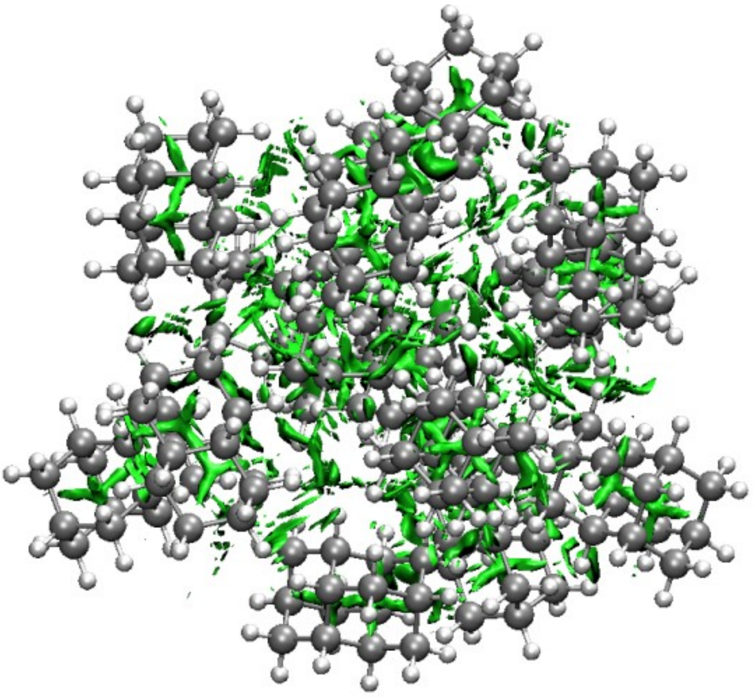

a) **b)**

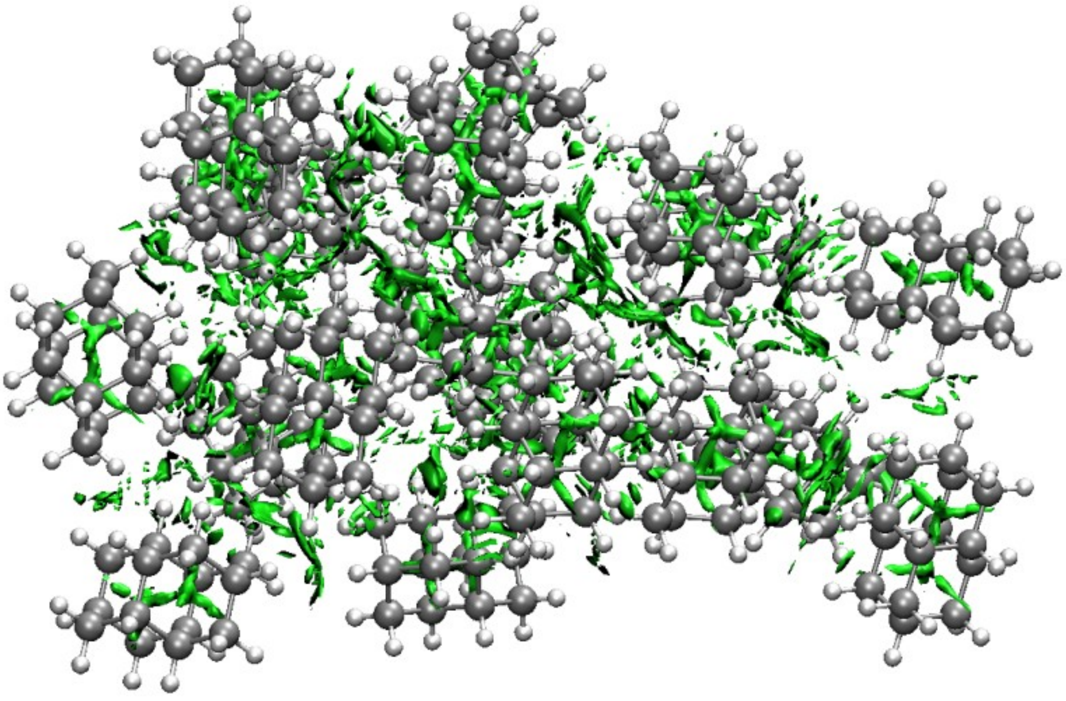

c)

Figure S4. NCI plots of **CL2** (a), **CL13** (b) and **CL19** (c). Non-covalent interactions are depicted in green.

Table S1. Electronic energies, zero-point vibrational energies, enthalpies and Gibbs energies of diamantane (**D**), 1-diamantyl cation (**Dp**) and clusters **CL2**, **CL13** and **CL19** in Hartree computed using the GFN2-xTB method at 0.4 K.

**compound**	*E*	*ZPVE*	*H*	*G*
**D**	-40.292908	0.312047	-39.980856	-39.980866
**Dp**	-39.689966	0.298400	-39.391561	-39.391573
**CL2**	-79.991445	0.609472	-79.381968	-79.381981
**CL13**	-523.327286	4.049740	-519.277541	-519.277484
**CL19**	-765.155096	5.925712	-759.229379	-759.229283

Table S2. Electronic energies of diamantane (**D**), 1-diamantyl cation (**Dp**) and clusters **CL2**, **CL13** and **CL19** in Hartree computed at various levels of theory at 0.4 K.[a]

**level of theory**	**D**	**Dp**	**CL2**	**CL13 s.p.**	**CL19 s.p.**
HF-3c	-538.455970	-537.596438	-1076.065555	-6999.239776	-10230.072868
PBEh-3c	-544.416185	-543.524277	-1087.954447	-7076.701972	-10343.290096
B3LYP-gCP-D3(BJ)/def2-TZVPP	-545.439573	-544.561400	-1090.015310	-7090.011461	-10362.742886
B3LYP-gCP-D3(BJ)-ABC/def2-TZVPP	-545.439079	-544.560961	-1090.013829	-7089.990531	-10362.711159
B3LYP-gCP/def2-TZVPP	-545.359585	-544.483153	-1089.844172	n.d.	n.d.
ωB97X-gCP-D3(BJ)/def2-TZVPP	-546.087719	-545.193132	-1091.292373	-7098.415323	-10375.033228
TightPNO-DLPNO-CCSD(T)/cc-pVTZ	-544.570843	-543.679302	-1088.261099	n.d.	n.d.

[a] Geometries for **CL13** and **CL19** single point computations from the GFN2-xTB level of theory optimization at 0.4 K.

Table S3. Electronic energies of diamantane (**D**), 1-diamantyl cation (**Dp**) and cluster **CL2** and interaction energies, ΔH(0 K), for **CL2** computed at various levels of theory with or without the geometrical counterpoise (gCP) correction.[a,b]

**Level of theory**	E(**D**) / Hartree	E(**Dp**) / Hartree	E(**CL2**) / Hartree	$\Delta H_{0\,K}$(**CL2**)[c] / kcal mol^{-1}
**B3LYP-gCP-D3(BJ)/def2-TZVPP**	-545.439573	-544.561400	-1090.015310	**-8.4**
**B3LYP-D3(BJ)/def2-TZVPP**	-545.463886	-544.584505	-1090.064070	**-9.5**
**ωB97X-gCP-D3(BJ)/cc-pVDZ**	-545.875011	-544.983341	-1090.867050	**-4.8**
**ωB97X-D3(BJ)/cc-pVDZ**	-545.965977	-545.070619	-1091.049375	**-7.7**
B3LYP-gCP-D3(BJ)/6-31G(d)	-545.173000	-544.302869	-1089.488033	-7.0
B3LYP-gCP-D3(BJ)/6-31+G(d,p)	-545.209420	-544.334072	-1089.554587	-6.3
B3LYP-gCP-D3(BJ)/aug-cc-pVDZ	-545.299474	-544.421622	-1089.739607	-11.0
B3LYP-gCP-D3(BJ)/cc-pVDZ	-545.269196	-544.392380	-1089.677294	-9.2
B3LYP-gCP-D3(BJ)/def2-TZVP	-545.432167	-544.554168	-1090.000545	-8.3
ωB97X-gCP-D3(BJ)/aug-cc-pVDZ	-545.899884	-545.008649	-1090.919261	-6.1
ωB97X-gCP-D3(BJ)/def2-TZVP	-546.081411	-545.186981	-1091.279850	-6.6
ωB97X-gCP-D3(BJ)/def2-TZVPP	-546.087719	-545.193132	-1091.292373	-6.6

[a] Interaction energies are defined as a difference between the energy of the cluster and the energy of the corresponding number of diamantane moieties. [b] Single point computations done on optimized geometries obtained by using the B3LYP-D3(BJ)/def2-TZVPP level of theory. [c] *ZPVE* taken from GFN2-xTB computations.

Table S4. Geometries of diamantane (**D**), 1-diamantyl cation (**Dp**) and clusters **CL2**, **CL13** and **CL19** in Cartesian coordinates in Å computed using the GFN2-xTB method at 0.4 K.

**D**			
1	-6.922650380	-0.687599871	4.116590698
6	-6.677725611	-0.933747254	5.157024171
1	-6.653967466	-3.058765143	4.791389859
6	-7.157481778	-2.354242769	5.464592076
1	-7.540260243	0.407161785	8.209362171
6	-7.036746086	-0.297360114	7.536159587
1	-7.271577855	-1.964002593	8.884161842
6	-7.516502511	-1.717855609	7.843728469
1	-7.124065008	-3.716549219	7.135804875
6	-6.794808065	-2.694480854	6.912099365
1	-7.070163953	1.064946675	5.864947186
6	-7.399420753	0.042878205	6.088652860
1	-9.164020666	0.202731758	4.855624949
6	-8.910339477	-0.055135796	5.886885386
1	-4.775378396	-3.306040713	6.456414908
6	-5.283891506	-2.596462489	7.113880905
1	-9.418857445	0.654440295	6.544351379
1	-5.030205842	-2.854328128	8.145141508
1	-9.002014744	-3.475582663	5.469132712
6	-8.668036277	-2.455852196	5.261876024
1	-4.828486243	0.179249390	5.121288754
6	-5.166631194	-0.833094929	5.356161183
1	-9.365745558	-2.830864648	7.879455369
6	-9.027595453	-1.818520522	7.644586006
1	-5.192206834	0.823987343	7.531609570
6	-5.526190947	-0.195742079	7.738867030
1	-9.537468441	-1.129373558	8.322416745
1	-5.275315220	-0.425788764	8.777369361
1	-8.918916936	-2.225804527	4.223374088
1	-4.656753837	-1.522241971	4.678332269
6	-4.819601035	-1.174909935	6.803686912
6	-9.374627389	-1.476692530	6.197063984
1	-3.735442411	-1.103081688	6.948071898
1	-10.458785986	-1.548520698	6.052678883

**Dp**			
1	-0.098069070	2.149599953	1.179912168
1	-0.098095734	-2.149541254	1.180016655
1	0.109104533	2.119139261	-1.289351102
1	0.089545733	0.000059807	2.446001576
1	0.109079661	-2.119204746	-1.289246192
1	-2.191033955	-1.274088608	-1.765294424
1	-2.155990224	2.145522986	-0.236064710
1	-2.155356981	-0.880184950	1.947203806
1	2.161974022	2.147860601	0.150652112
1	2.198176185	-1.289318428	1.680727889
1	2.095944644	-0.892843494	-2.026946755
1	-2.156017184	-2.145509570	-0.235961711
1	-2.191020931	1.274030080	-1.765356148
1	-2.155348555	0.880304366	1.947162614
1	2.198193908	1.289374830	1.680665724

1	2.161948506	-2.147879807	0.150756788
1	2.095955785	0.892718027	-2.026991875
6	1.745215842	-0.000047554	-1.512935617
6	1.804663274	1.253112699	0.663329528
6	1.804647307	-1.253102308	0.663389409
6	-1.809630130	1.244727147	-0.743499851
6	-1.790624880	0.000045521	1.416503255
6	-1.809644806	-1.244742090	-0.743438839
6	2.311578637	-0.000014710	-0.037252102
6	-2.313257004	0.000013294	-0.019393731
6	0.282959237	-1.240417601	0.708047202
6	0.319396781	-0.000032443	-1.238334120
6	0.282974901	1.240449016	0.707986337
6	-0.259763066	0.000036289	1.408619555
6	-0.283565287	-1.236938358	-0.780078239
6	-0.283550610	1.236903129	-0.780138546
1	3.401385173	-0.000022981	-0.104005386
1	-3.405472077	0.000020130	-0.008525328

**CL2**			
1	1.370489985	-0.650040867	-6.404975478
1	1.635387689	1.782585310	-6.004025242
1	0.175768379	-1.204258134	-2.338796875
1	0.425318758	1.256181763	-1.934559012
1	1.426666223	2.663458347	-3.697990180
1	0.359143062	-2.075817725	-4.648926779
1	2.821540772	-1.982236356	-4.809876471
1	-0.553921786	2.775320994	-5.186636109
1	2.318202127	-2.196923974	-3.133711563
1	-1.034986814	2.561295139	-3.504809982
1	3.604210929	1.658308760	-4.505279877
1	-1.092928392	-0.752340959	-6.251779255
1	2.884017518	1.343388708	-2.106523562
1	-1.771645359	-1.133228672	-3.899758509
1	2.359781371	-0.244034167	-1.543093920
1	-1.893009762	0.195359851	-2.712535769
1	3.577626503	0.292347841	-5.620029243
1	-0.575908244	0.850907902	-6.778855373
6	-0.564622122	0.151554294	-5.941840882
6	3.065727852	0.746874926	-4.770235069
6	-1.249740259	-0.214373868	-3.620847297
6	2.357193840	0.434087946	-2.399746464
6	-0.538902734	2.076199942	-4.349406360
6	2.320747048	-1.496090201	-3.971181665
6	-1.278726732	0.797945058	-4.742169645
6	3.062795477	-0.218550660	-3.586419636
6	0.164280467	-0.496471129	-3.175219794
6	0.920302559	0.778310543	-2.788779626
6	0.871192771	-0.181980453	-5.550879209
6	1.627711382	1.089344780	-5.156757637
6	0.904497896	1.742756085	-3.976163350
6	0.887404501	-1.156024346	-4.373485625
1	-2.308406942	1.030609991	-5.031285298
1	4.091732513	-0.463844306	-3.309733865
6	-5.137073329	1.667221367	-1.884344105

6	-5.833085207	0.364841568	-1.507172305
6	-4.879785748	-0.508069423	-0.699198363
1	-5.803815329	2.297790396	-2.477374329
1	-6.722243646	0.580620271	-0.913434976
1	-6.160034665	-0.163158937	-2.405065671
1	-5.368197203	-1.450524888	-0.436491159
6	-4.678104267	2.406768698	-0.633155489
6	-3.890778572	1.331617811	-2.743751473
6	-3.620560153	-0.849777961	-1.550915409
6	-4.419665099	0.212487649	0.566990854
6	-3.733910946	1.518602509	0.168973551
1	-4.179723931	3.338914723	-0.906486384
1	-5.542596829	2.666306868	-0.020798412
6	-3.027435260	0.479898411	-1.878430426
1	-4.212349618	0.801883165	-3.641183602
1	-3.396145513	2.259190858	-3.032534527
6	-2.680600043	-1.737943023	-0.736828663
1	-3.949222486	-1.369853890	-2.456077361
6	-3.471210008	-0.673082539	1.374088142
1	-5.295188015	0.445481463	1.180428188
6	-2.471670437	1.174138094	-0.681277668
1	-3.392048880	2.048589771	1.062408737
6	-2.243384438	-1.001784927	0.528070502
1	-3.197813305	-2.660367698	-0.470081761
1	-1.811421123	-2.011073729	-1.337801383
1	-3.168831688	-0.157068378	2.286435560
1	-3.981851567	-1.591747165	1.665988574
6	-1.533729746	0.294600179	0.144736405
1	-1.976573323	2.110454785	-0.957120716
1	-1.561317253	-1.634541939	1.101796781
1	-0.628190921	0.074094290	-0.421495838
1	-1.234565601	0.835281761	1.043718097

**CL13**			
1	-0.274923490	0.474360982	5.393782943
1	-3.405945235	-0.752859755	-7.737089844
1	2.638521151	-3.129784741	-0.820424186
1	4.651346118	-1.432576192	-4.196491617
1	6.117585550	-0.703422513	-0.238979258
1	1.103372244	2.395373106	6.131839500
1	-1.774840325	-0.478774357	-9.584742008
1	2.939631900	-2.970431446	-3.279571385
1	6.412114203	-0.537822195	-2.695537873
1	4.402799647	-2.243630225	0.679000107
1	-3.785483184	2.418546993	6.887041803
1	-0.608997492	0.998037324	-5.008881666
1	-2.410428260	4.338295325	7.621265157
1	1.021600621	1.270335112	-6.855746936
1	0.061829154	4.184370553	7.498009190
1	0.534622668	0.261305897	-9.066652737
1	-2.749029495	0.627176245	5.508588023
1	-2.922852925	0.264800599	-5.531035975
1	-1.542477773	-0.527864597	7.336681946
1	-2.430286124	-2.140959194	-5.880306811
1	2.417852391	-0.844363993	0.209260178

1	2.379331673	-0.568811930	-3.515918560
1	5.029580462	1.166220318	-1.545089030
1	0.292591637	4.537225140	5.056478556
1	-1.114481286	1.940899797	-9.841205401
1	5.202706216	-3.836679965	-3.961091757
1	7.502503048	-2.404485526	-1.394052527
1	4.626454439	-4.536810546	-0.339291717
1	3.852907333	0.161452583	0.444392273
1	1.553121366	-1.280239389	-2.121220825
1	4.424960826	0.860486512	-3.177657640
1	-2.989145003	0.280278753	7.947417830
1	-1.272387285	-1.424616044	-4.751760840
1	-1.158079765	5.334182996	5.672463764
1	0.037508112	2.664679927	-8.716369105
1	6.638274223	-2.836558530	-3.722685544
1	6.676408875	-3.105758729	-0.000305392
1	4.024060778	-4.841188204	-1.971996960
1	0.870930982	2.040432826	8.573471286
1	-0.126646581	-2.163691550	-8.814877394
1	-1.488357390	1.611188286	3.567387429
1	-3.912907185	1.649407940	-7.389534307
1	-1.196756546	3.186600622	9.451210003
1	1.519553847	-1.133612407	-7.205628968
1	-3.569691414	2.761830464	4.443835525
1	-2.261804852	2.684060651	-5.777610043
1	-2.785515237	2.461245573	9.193527979
1	1.045656953	-0.830634366	-5.531745893
1	-3.420796360	4.292197316	5.312158093
1	-0.634580415	3.096789379	-6.334885308
1	0.731811852	0.513519268	7.698306298
1	-1.753042312	-2.579568495	-8.268520912
1	0.100793076	2.351103915	3.818838991
1	-3.434969169	1.342628749	-9.060443551
6	-0.886641308	2.202902328	4.266018688
6	-3.076806721	1.355332386	-8.029169916
6	4.499542628	-4.094813477	-1.330768067
6	5.669024142	-3.104966931	-3.297150717
6	6.524050938	-2.679439639	-0.994666225
6	0.245148310	1.474938199	7.880104516
6	-0.941169840	-1.857084709	-8.155398766
6	2.531103108	-0.998165414	-2.521658055
6	4.553219624	0.419574244	-2.185461647
6	3.386442468	-0.571889242	-0.218295576
6	-2.936004430	3.330621189	5.132612924
6	-1.448332004	2.375849008	-6.440801459
6	-1.798719866	2.607132917	8.748250249
6	0.686003279	-0.839087146	-6.564874627
6	-0.694935026	4.361734531	5.491028039
6	-0.781046635	1.945821862	-8.801475411
6	-1.998581557	0.448614462	7.518748828
6	-1.608463099	-1.425949024	-5.793197715
6	-1.553952650	3.552894505	4.521351804
6	-1.933975363	2.358084849	-7.888626982
6	5.860019294	-3.708538578	-1.907100855
6	-1.138149962	1.254490701	8.489369618

6	-0.457910211	-1.840867033	-6.707454425
6	3.194406261	0.030060994	-1.608194074
6	-2.792561468	2.570898905	6.449205831
6	-0.957785989	0.983103497	-6.050640329
6	5.638516341	-1.439194577	-0.896078403
6	3.617305503	-2.852259168	-1.234350305
6	4.786426251	-1.862852159	-3.196830952
6	-1.933631548	3.369561460	7.431827442
6	0.197160699	0.553932484	-6.958388547
6	5.437955589	-0.822140041	-2.282701248
6	4.270556307	-1.812818501	-0.320550230
6	3.418904447	-2.236519934	-2.621546873
6	-0.751290535	1.442669971	5.583733824
6	-2.583285598	-0.035653351	-7.637106453
6	0.107950817	2.240864215	6.566128180
6	-1.428378262	-0.463114868	-8.545471550
6	-0.555228293	3.599165750	6.807809989
6	-0.291751948	0.552957648	-8.408830325
6	-2.131481494	1.212536346	6.204189786
6	-2.093697836	-0.032787073	-6.187352288
1	-1.651435094	4.105962606	3.576375462
1	-2.285085642	3.357493195	-8.168897161
1	6.493353896	-4.598934726	-1.977831978
1	-1.040096142	0.707251799	9.432598375
1	-0.108126985	-2.840193515	-6.426844042
1	2.559760557	0.923518948	-1.534532473
1	-0.900244191	-3.152407127	2.010165959
1	1.832135011	-7.216194842	-3.015547782
1	0.737395634	2.221348022	-1.604867820
1	0.254262663	-5.289648884	2.455238041
1	-0.197188123	-8.635194775	-3.150823754
1	-1.202030425	3.316057924	-2.740715595
1	-0.048062525	-1.863847386	6.007639703
1	-0.611548678	-3.792727286	-2.240097901
1	2.043707235	6.032770615	-0.172174988
1	1.108472074	-4.007509001	6.449028024
1	-2.647385882	-5.214017148	-2.362968926
1	0.125113943	7.121306912	-1.305554897
1	0.993496040	-5.821938779	4.761315761
1	-2.433960827	-7.566439284	-3.111049748
1	-1.317313636	5.779105094	-2.811157494
1	-0.782870826	-1.325308960	3.699367317
1	1.617229113	-4.863496757	-2.262048666
1	2.167564879	3.557323578	-0.086313907
1	-2.796930915	-2.785924725	3.660673156
1	1.718337393	-5.334020944	-4.696608984
1	2.903536531	3.175277119	-2.421775138
1	2.640305550	-4.890033325	3.159232865
1	-1.514224199	-8.500756542	-1.006350450
1	-2.598858440	4.572613007	-1.076616358
1	-2.428847705	-2.258308376	5.305574088
1	0.707813141	-3.921761573	-4.367739859
1	3.450454140	4.744094995	-1.832384368
1	2.997369487	-4.364514791	4.806531633
1	-2.529430618	-7.093196261	-0.681336346

1	-2.058112908	6.139815855	-0.473785637
1	-1.399331556	-6.224188767	4.046293507
1	-1.109685812	-7.703330305	-5.258267311
1	0.084119747	4.502864291	-4.473703352
1	1.099543966	-1.667681790	2.073468429
1	1.743985234	-6.732549912	-0.586533059
1	0.074828016	2.631356113	0.768944301
1	-0.891569404	-5.472283108	6.402365361
1	-2.551466461	-5.690008054	-4.794806537
1	0.866568984	6.747818587	-3.640158587
1	1.608263430	-0.920933165	4.414161119
1	0.301671788	-4.719010149	-0.126441773
1	0.765796340	4.855372199	1.588602550
1	-1.319631827	-3.839540523	6.918611106
1	-1.800237979	-4.130264437	-4.428508898
1	2.250387161	6.851704161	-2.551276879
1	2.381809652	-2.032129088	5.547172327
1	-1.460416223	-4.869981884	-0.158682737
1	-0.093485937	6.306618699	1.068091579
1	-2.174092192	-5.111952144	2.913442246
1	0.649110895	-7.558017243	-5.216909650
1	0.924641657	3.030914416	-3.979140864
1	1.530944998	-3.300579178	1.543826732
1	0.994772052	-8.290967346	-0.944580782
1	-1.373360331	2.397522500	-0.240830418
6	1.176444766	-2.711635227	2.393508192
6	0.892835161	-7.211928483	-1.077379992
6	-0.464632046	3.113068190	-0.050851405
6	-1.467202254	-5.176828530	3.745060873
6	-0.276581874	-7.203908590	-4.758236247
6	0.613443815	4.024672209	-3.648158954
6	1.674857278	-1.970112291	4.717305271
6	-0.533974523	-5.220326713	-0.622802332
6	0.241643798	5.318385152	0.749672142
6	-0.968688884	-4.434490941	6.071898681
6	-1.702642099	-5.211764542	-4.300048928
6	1.382986663	6.246193420	-2.819738083
6	2.279432547	-4.276832452	3.988488241
6	-1.590476866	-7.419047907	-1.134556585
6	-1.717500929	5.153277505	-0.791723661
6	-2.071361443	-2.871357872	4.474899258
6	0.780349269	-5.006141588	-4.242468215
6	2.567600297	4.167317921	-2.113014715
6	2.169626854	-2.818954601	3.548126876
6	-0.412462638	-6.732322039	-0.446587016
6	-0.981258938	4.473668512	0.361600892
6	-1.960657655	-4.329143751	4.915940796
6	-0.397685542	-5.691530104	-4.931940355
6	1.834568566	4.855418506	-3.261583716
6	0.304726446	-2.473777635	5.166982739
6	-0.529850547	-4.880048834	-2.111449641
6	1.169732066	5.437687029	-0.455304779
6	0.400417585	-3.933484752	5.615998120
6	-1.707607919	-5.559667471	-2.812967424
6	0.446973620	6.122597633	-1.618752310

6	-0.190849949	-3.218511542	2.847067375
6	0.894106247	-6.870057842	-2.566241658
6	0.398046422	3.214371660	-1.288378366
6	-0.095489860	-4.678332168	3.295108185
6	-0.283111555	-7.549317957	-3.270159351
6	-0.323741685	3.904798390	-2.448332964
6	0.909681329	-4.776563615	4.444481806
6	-1.585654272	-7.073246459	-2.622322144
6	-0.782485536	5.289840143	-1.990984223
6	-0.701604168	-2.374486403	4.017318899
6	0.772763857	-5.357064690	-2.756436275
6	1.637417651	4.053911089	-0.907348066
1	3.150562503	-2.456859505	3.220563087
1	-0.418613623	-6.978692216	0.621587881
1	-1.640429287	4.388044395	1.231924296
1	-2.943622882	-4.686415642	5.242241506
1	-0.394486610	-5.443416057	-5.999446340
1	2.504603870	4.942677872	-4.121593493
6	-4.479005675	1.183795245	1.328062592
6	-5.202130551	0.822908001	0.037466545
6	-4.227053001	0.159952135	-0.926776449
1	-5.162466843	1.680589364	2.021067474
1	-6.021166165	0.135524676	0.252571709
1	-5.636395405	1.717389161	-0.412453370
1	-4.737021232	-0.089098129	-1.861696062
6	-3.880290530	-0.061780608	1.967877176
6	-3.331283880	2.171022316	0.984896840
6	-3.059406874	1.137534537	-1.276590695
6	-3.627661927	-1.100279960	-0.308286838
6	-2.918655722	-0.723722310	0.990521900
1	-3.359295715	0.196545619	2.892781042
1	-4.676399243	-0.760882989	2.228539176
6	-2.449047366	1.429910978	0.046929473
1	-3.758270200	3.065542321	0.530490467
1	-2.812757910	2.446752486	1.903415009
6	-2.103036569	0.450905790	-2.248462086
1	-3.485947097	2.036902484	-1.728790883
6	-2.654296782	-1.773320917	-1.273045804
1	-4.437602068	-1.797896043	-0.077830719
6	-1.756195514	0.255390349	0.643545146
1	-2.472856342	-1.611551097	1.447905840
6	-1.522707771	-0.804331032	-1.604158791
1	-2.643201657	0.187426807	-3.158815087
1	-1.300782022	1.134449088	-2.530662205
1	-2.250722626	-2.677670368	-0.815818143
1	-3.174573881	-2.066900138	-2.185850568
6	-0.788212989	-0.426952206	-0.320499863
1	-1.242816012	0.522917752	1.572222687
1	-0.823684236	-1.279131919	-2.298892194
1	0.049042421	0.238175894	-0.540106612
1	-0.383633775	-1.323658338	0.151178100
1	-5.851599278	7.354424282	-4.017173826
6	-5.955638015	6.263879773	-3.989411051
1	-5.332232185	0.904861904	6.398287643
6	-6.219484122	0.374776602	6.766154294

1	-6.065027538	6.082254723	-6.135335111
6	-6.661729542	5.791694458	-5.262823615
1	-5.095323088	-1.361508913	7.376924593
6	-5.951475917	-1.131271013	6.731539992
6	-6.930318603	4.346208013	-2.740100154
1	-7.525448252	4.055192613	-1.864701927
1	-9.496046787	0.184353337	5.722193356
6	-8.640589809	-0.045388830	6.369013613
1	-7.738894936	2.780756295	-3.983210416
6	-7.636112526	3.872240323	-4.012166748
1	-9.259075584	-2.084809349	6.692745842
6	-8.371669992	-1.551730407	6.334037725
1	-7.275079142	3.918232341	-6.137129208
6	-6.783929042	4.266397957	-5.221558091
1	-6.998944981	-2.944497032	7.243743443
6	-7.188776390	-1.864609039	7.253893331
1	-6.309808829	6.214039302	-1.862815847
6	-6.805930316	5.870200387	-2.779136662
1	-7.592872750	1.767926035	5.858563776
6	-7.402780472	0.687295057	5.847697920
6	-8.191092004	6.505303039	-2.874442465
1	-8.099155798	7.593654958	-2.885805283
1	-6.202552876	0.780731373	4.058037381
6	-7.085440210	0.245510125	4.420888397
1	-4.801558003	3.907345594	-5.997055384
6	-5.398049407	3.630682123	-5.124511689
1	-6.656958979	-1.659121558	9.335515459
6	-7.498834089	-1.419528871	8.681545924
1	-8.786993266	6.228019508	-2.001056726
1	-7.921374655	0.490959117	3.760999315
1	-5.490516152	2.541226484	-5.112902080
1	-8.374399895	-1.955342061	9.054108606
1	-8.538946625	6.100933312	-6.280270566
6	-8.045923471	6.429469750	-5.362833965
1	-5.422999557	-2.645969714	5.289136286
6	-5.635651372	-1.574698502	5.305069541
1	-4.066524769	5.983186755	-2.981293900
6	-4.568827567	5.630681059	-3.886952900
1	-6.700607830	1.901989150	8.214396521
6	-6.526164428	0.823192411	8.192787539
1	-9.524485740	4.154966821	-5.014697788
6	-9.021232309	4.507158876	-4.111648983
1	-7.885854762	-3.076129964	4.886576189
6	-8.061391323	-1.997443868	4.906857937
1	-5.057621639	4.039048537	-1.717518364
6	-5.546892274	3.707715564	-2.640433459
1	-9.156706179	1.475341689	7.811289927
6	-8.951620530	0.402137096	7.795717590
1	-9.629604805	4.210548429	-3.253601691
1	-8.914253478	-1.784118627	4.258060373
1	-5.652108860	2.618736072	-2.607969396
1	-9.843116121	-0.113697809	8.158467922
1	-7.950104604	7.516636070	-5.405737879
1	-4.744076348	-1.053214467	4.945792800
1	-3.961050512	5.926538274	-4.745073644

1	-5.672723448	0.609101137	8.841389096
6	-4.706289281	4.110419520	-3.850390303
6	-7.762908837	0.084806473	8.701104452
6	-8.882666728	6.027525602	-4.149885157
6	-6.824877940	-1.258730857	4.400427931
1	-3.708702794	3.653618404	-3.779869319
1	-7.985699227	0.404995108	9.724321869
1	-9.875194396	6.483993561	-4.220843200
1	-6.604745323	-1.579226657	3.373107523
1	-9.038209025	-2.146200948	-3.520819239
6	-8.104483541	-2.499166163	-3.974498080
1	-6.953026783	-6.397942325	3.607894894
6	-6.642809860	-5.981093255	2.643019892
1	-5.064077037	4.073739425	2.725951440
6	-5.978584444	4.680894056	2.678648823
1	-6.866120850	-1.603036275	-2.452617577
6	-6.956738061	-1.580443932	-3.547909924
1	-8.329324864	-4.640461534	2.524899281
6	-7.870562147	-5.428445623	1.914758918
1	-6.091884790	4.613838328	0.524339639
6	-6.055601821	5.374334292	1.316693160
1	-7.033748574	-2.944470896	-7.214751086
6	-6.941601952	-2.963364275	-6.123000818
1	-5.111137241	-7.276056974	-0.155219988
6	-5.571026279	-6.491019430	0.456602135
1	-7.170116089	7.342162850	4.508395049
6	-7.199847767	6.584210866	3.717712570
1	-4.861749670	-2.396348694	-6.154927790
6	-5.794952744	-2.043427245	-5.699388016
1	-6.481391224	-5.524618123	-1.237419386
6	-6.797652074	-5.940074871	-0.273075131
1	-8.184288901	7.889244153	2.312032003
6	-7.275963205	7.278488138	2.355673011
1	-4.824198490	-1.444172911	-3.873990631
6	-5.658644051	-2.094004789	-4.175810087
1	-8.296898748	-4.422061717	0.058097619
6	-7.422712547	-4.832375810	0.578753428
1	-7.404258666	6.701741010	0.283111541
6	-7.335621301	6.211224689	1.260959936
1	-9.070016904	-3.094038974	-5.805929355
6	-8.240219619	-2.448694518	-5.497235536
1	-5.146098948	-7.498666459	2.315294740
6	-6.018710760	-7.089577641	1.791866306
1	-5.850277655	5.256318926	4.750580773
6	-5.918767262	5.748928774	3.773164839
1	-9.453578454	-0.663193656	-5.505911306
6	-8.519991949	-1.016903065	-5.950019735
1	-7.338870947	-8.637492178	2.508771333
6	-7.034461476	-8.204698250	1.553604546
1	-3.782269512	6.045399518	3.625720514
6	-4.696728893	6.643172374	3.570121115
1	-5.275451450	-3.547770329	-2.624125894
6	-5.380354638	-3.522657374	-3.713496242
1	-6.868031957	-2.920845599	1.410775820
6	-6.409331096	-3.714876434	0.814653221

1	-8.596378594	4.562893109	0.669884648
6	-8.555121059	5.314802197	1.461366904
1	-8.632317822	-0.985846826	-7.035723225
1	-6.578788580	-8.998259963	0.957209207
1	-4.653336044	7.394688027	4.360982649
1	-4.443844946	-3.879806697	-4.148166923
1	-6.110627628	-3.286128084	-0.148053194
1	-9.467049097	5.912095163	1.402935154
1	-6.408708493	0.504468988	-3.687113515
6	-7.229861115	-0.148621699	-4.002126019
1	-9.767064568	-6.136030462	1.168894757
6	-8.888606638	-6.541222255	1.676857380
1	-4.876649614	6.742184498	0.130732419
6	-4.831773818	6.264545626	1.112990990
1	-8.659956476	-4.577041285	-3.808377603
6	-7.832272037	-3.928655065	-3.513786882
1	-4.758123565	-5.262855255	3.412728587
6	-5.626549719	-4.866835857	2.881080625
1	-7.129753718	3.279158597	3.848155765
6	-7.197621359	3.783530547	2.881557088
1	-5.239127031	0.035924990	-5.866505041
6	-6.070274230	-0.612396389	-6.156672556
1	-8.679348141	-6.653933822	-1.048994056
6	-7.814298537	-7.052813358	-0.514077683
6	-6.056431514	8.174430785	2.148442652
1	-6.125064055	8.676037541	1.180201959
1	-7.477201341	-5.049252514	-5.981657895
6	-6.663371687	-4.394024663	-5.664603633
1	-3.673932028	-5.776873036	1.196135918
6	-4.554909692	-5.374489040	0.688977092
1	-8.367325571	5.207434209	4.902073410
6	-8.420619243	5.688989273	3.922734000
1	-6.151855761	-0.579768613	-7.244936292
1	-7.367771452	-7.832767426	-1.134935126
1	-6.025213160	8.944114186	2.922268838
1	-5.741873692	-4.758277179	-6.125288661
1	-4.232085012	-4.961191745	-0.271990506
1	-9.330324224	6.292130521	3.896033223
1	-8.146479512	0.218486250	-3.536315857
1	-9.215653085	-6.953204622	2.634119382
1	-3.921318885	5.658495534	1.152459551
1	-7.757945546	-3.957040638	-2.423947767
1	-6.072250239	-4.084623034	3.499102406
1	-7.230587673	3.015767464	2.101936615
6	-6.533301336	-4.427694461	-4.143291616
6	-5.189944536	-4.278780671	1.541600464
6	-8.467301999	4.630256066	2.823555484
6	-7.366300659	-0.113052824	-5.522701436
6	-8.252907235	-7.639412623	0.826078431
6	-4.786989425	7.328142892	2.208112856
1	-6.335225109	-5.454079548	-3.814681943
1	-4.457774531	-3.478552748	1.718076028
1	-9.342626269	3.987546404	2.968940944
1	-7.563878474	0.914135865	-5.850380013
1	-8.981759721	-8.438681167	0.655695964

1	-3.910249815	7.969304499	2.060598631
6	-9.962427407	-1.292176182	1.214879700
6	-10.921860254	-1.875310575	0.180472686
6	-11.472712765	-0.749997835	-0.693304097
1	-9.565820788	-2.099145286	1.842419480
1	-10.398961607	-2.606267693	-0.442053656
1	-11.742456702	-2.390641661	0.683875891
1	-12.160958561	-1.172236981	-1.434448263
6	-8.810077903	-0.591730157	0.498444387
6	-10.706117965	-0.279735639	2.083569025
6	-12.220938450	0.273044964	0.164627149
6	-10.327749758	-0.039845568	-1.418322395
6	-9.360555752	0.531030392	-0.378837861
1	-8.115831305	-0.180514444	1.238318992
1	-8.266432953	-1.311799206	-0.119735586
6	-11.253917869	0.843370545	1.204860954
1	-11.526866999	-0.772989715	2.608777638
1	-10.030593058	0.136671496	2.836111592
6	-12.771874654	1.394530312	-0.713911220
1	-13.050865756	-0.224740867	0.678762543
6	-10.875653765	1.078914056	-2.301712983
1	-9.793574145	-0.768023795	-2.040044893
6	-10.108862383	1.553702641	0.478602723
1	-8.529097289	1.029916277	-0.895653002
1	-11.784803242	1.567687840	1.833554579
6	-11.619615545	2.091554414	-1.433749603
1	-13.472007301	0.983204894	-1.444318827
1	-13.315604755	2.114567105	-0.098195053
1	-10.057056297	1.574897036	-2.831224654
1	-11.552667896	0.661607572	-3.050506693
6	-10.656132208	2.674741166	-0.402480652
1	-9.420139693	1.978126384	1.220456960
1	-12.013286564	2.896305759	-2.064478640
1	-11.170722194	3.414305519	0.214882327
1	-9.831332384	3.179880751	-0.913782305
**CL19**			
1	-0.693050675	2.068312831	6.036399812
1	-2.688886346	0.244499457	-6.883444992
1	4.759402198	0.334177766	-1.638958359
1	4.609643879	-3.189711492	0.786837066
1	1.984404123	-2.790396041	-2.571313037
1	-1.447342730	4.436767083	6.133328024
1	-0.902634627	0.675359856	-8.544776248
1	5.722674935	-1.059347523	0.169040893
1	2.942697708	-4.180871301	-0.759486499
1	3.095360166	-0.658692436	-3.187002555
1	-1.667876493	1.591155128	10.174865392
1	0.212743382	0.337796935	-3.734791959
1	-2.414412908	3.955093151	10.274889788
1	1.998685472	0.756766664	-5.395034430
1	-2.018493984	5.436338543	8.326264458
1	1.467795018	0.636078159	-7.813075239
1	-1.093668055	0.590206488	7.984143113
1	-2.162097731	0.380525859	-4.465391205
1	1.134853115	1.646093112	7.709207815

1	-2.207051414	-1.841835960	-5.560554033
1	2.285996870	0.694942814	-1.285738026
1	3.778011905	-0.986732909	1.704265101
1	1.058778027	-2.663785301	-0.226279449
1	-3.844314351	4.581620915	6.887610363
1	0.292258239	2.809707917	-8.025024269
1	6.559575294	-3.258219830	-0.741473282
1	3.867031104	-4.306556479	-3.104309812
1	5.564408255	-1.017110486	-3.558111066
1	1.144079632	-0.603908575	-1.659161214
1	3.845000916	0.464903696	0.697715502
1	2.147574640	-2.828054728	1.156661583
1	0.734819228	1.443240356	9.417253481
1	-1.006800294	-1.811774066	-4.266213947
1	-4.245294246	4.381387971	8.596478624
1	1.501035095	2.870566897	-6.744219291
1	5.413396932	-4.545051671	-1.126357256
1	3.930931508	-2.850568896	-4.103846795
1	6.647352663	-1.177237706	-2.172627129
1	0.381766648	5.296559605	7.572412655
1	0.249340052	-1.512121821	-8.352625355
1	-2.930441419	1.019116609	6.286597491
1	-2.658450652	2.466276694	-5.780688852
1	-0.187888727	5.011753007	10.013362682
1	1.957677067	-1.461493135	-6.499100938
1	-3.488560139	0.726860909	8.735397723
1	-0.927338604	2.531786330	-3.923552528
1	-0.044039114	3.424892144	10.773181154
1	1.439331072	-1.586153660	-4.817467509
1	-4.041204186	2.112285549	9.681425433
1	0.785393089	2.678850732	-4.330286846
1	0.934184816	3.907486828	6.629622638
1	-1.466681370	-1.668915260	-7.968463601
1	-3.075628712	2.617935046	5.539855812
1	-2.140574894	2.592409478	-7.462799293
6	-2.809003164	2.091928547	6.462810198
6	-1.864954780	2.194985593	-6.483542039
6	5.646122780	-1.409770601	-2.541937581
6	5.558642157	-3.462360330	-1.127918986
6	4.030617131	-3.226698231	-3.082974006
6	0.261637811	4.219695067	7.433327541
6	-0.521595930	-1.237204660	-7.629941670
6	3.679696437	-0.616039378	0.680797273
6	2.062629691	-2.435004467	0.141551245
6	2.146105239	-0.389672876	-1.273930886
6	-3.371388834	1.804915886	8.875355877
6	-0.165852726	2.249435106	-4.655550292
6	-0.303911628	3.938694124	9.845257016
6	1.166440626	-1.187278323	-5.798039056
6	-3.576237299	4.045308169	7.801413126
6	0.549462695	2.421303028	-7.036939626
6	0.464969039	1.981054269	8.506091876
6	-1.252577819	-1.408800801	-5.252811570
6	-3.736474465	2.541724039	7.589143619
6	-0.536511477	2.798708655	-6.032291187

6	5.428588266	-2.921380351	-2.550161171
6	0.625108127	3.484490255	8.721767786
6	-0.161979022	-1.786875623	-6.252059161
6	2.281556208	-0.923966679	0.150199936
6	-1.925598086	2.121469027	9.251307387
6	-0.048570795	0.728192434	-4.727416059
6	2.987771771	-2.566112439	-2.185834124
6	4.600424541	-0.751492134	-1.645138442
6	4.513485210	-2.799392168	-0.233396537
6	-1.750279345	3.626323335	9.467590436
6	1.042332925	0.333299594	-5.724432147
6	3.105977997	-3.097063253	-0.755770414
6	3.193584430	-1.050504893	-2.167115176
6	4.721169450	-1.282915824	-0.215365155
6	-1.361501211	2.400096401	6.842186002
6	-1.735184713	0.675193868	-6.557733563
6	-1.185392435	3.905262750	7.058540330
6	-0.642632302	0.283154815	-7.554617427
6	-2.128909542	4.356060645	8.176759814
6	0.681362006	0.900931672	-7.099103970
6	-0.983105282	1.670451347	8.133481127
6	-1.373856643	0.111711501	-5.181680898
1	-4.774022459	2.315772447	7.318627234
1	-0.626991672	3.889653531	-5.978286954
1	6.177454022	-3.395953830	-3.193012981
1	1.663110271	3.708244048	8.989928531
1	-0.075223091	-2.877384387	-6.304085178
1	1.533485556	-0.448996756	0.800535549
1	-0.291487103	-1.978070342	2.622636817
1	0.323672569	-6.923965407	-4.558255671
1	0.372657403	1.889435865	-0.124604146
1	1.713987521	-3.169196823	3.440737037
1	-0.704648010	-8.428540638	-2.878489190
1	-1.332177849	3.704515503	-0.371878165
1	-2.519534698	-3.442348413	5.979469384
1	-2.438990496	-3.835727479	-3.489920495
1	2.373623423	4.185155675	2.873042830
1	-0.507559512	-4.644068720	6.786807632
1	-3.463634720	-5.335796995	-1.806494954
1	0.697591729	5.994216180	2.620152323
1	1.512964326	-4.731978851	5.350274099
1	-2.781377878	-7.721491254	-1.723337795
1	-0.975696680	5.853386120	0.796987395
1	-2.321233928	-1.876596964	4.079929441
1	-0.356672869	-4.542707256	-4.638761105
1	2.027150857	2.026066874	1.707218274
1	-2.209641762	-3.576232931	2.262415758
1	-1.400689334	-6.131593002	-6.232247453
1	2.673884364	2.766217411	-0.569191481
1	2.319930430	-2.426822038	5.768851017
1	-0.607342166	-7.397319665	-0.577546419
1	-2.462948635	4.178683908	1.829582147
1	-3.127044448	-4.180478688	3.651632112
1	-2.536281158	-4.858733419	-5.780697767
1	3.507184903	3.710914287	0.663418650

1	1.402535587	-3.029543041	7.150915177
1	-1.747844044	-6.125332349	-0.133116552
1	-1.617450343	5.098984113	3.073862388
1	0.908174869	-5.475460172	3.012074024
1	-2.876223272	-8.758731220	-4.027421908
1	0.149054888	5.376914989	-1.418205086
1	-0.417480880	-0.262694747	4.432041819
1	1.361891006	-5.319465161	-2.979234151
1	-0.233430342	1.139463659	2.177397744
1	-0.400353360	-6.345528693	4.985658623
1	-4.501563987	-6.946247831	-3.389035434
1	1.344507423	6.736998638	0.346352230
1	-1.721973604	-1.135430924	6.392328422
1	-0.271205167	-3.500754471	-2.334758977
1	0.901240851	2.498000011	3.919148131
1	-2.062149902	-5.810549657	5.247542231
1	-4.368401715	-5.341093487	-4.117854132
1	2.727942871	6.051402087	1.199428531
1	-0.972569904	-2.269884065	7.520247648
1	-1.556052057	-3.850154283	-1.168551468
1	0.330385462	4.129586792	4.284148722
1	0.161470501	-4.335678536	1.890520923
1	-1.593419986	-8.424721853	-5.193053560
1	0.698192252	3.743058995	-1.792771965
1	1.250238179	-0.794277043	4.163188741
1	1.226815512	-6.922388017	-2.249653892
1	-1.688369687	1.722729328	1.322797608
6	0.267436340	-1.113544314	4.520866148
6	0.653474616	-6.065671752	-2.610717387
6	-0.654185347	2.060332109	1.764740708
6	0.214173592	-4.635914736	2.940516879
6	-2.183999789	-7.991808411	-4.382520877
6	0.580455196	4.471667847	-0.987136961
6	-1.023587596	-1.973225027	6.470824050
6	-0.960058529	-4.273994512	-1.983029744
6	0.470763296	3.402522591	3.483336323
6	-1.078420560	-5.495108313	4.892025125
6	-3.794620452	-6.197472125	-3.755515954
6	1.759964078	5.813747694	0.754596682
6	1.323850704	-2.723901699	6.105820262
6	-1.156329910	-6.533426335	-0.957824368
6	-1.482529953	4.370382192	2.272224971
6	-2.132246972	-3.884015865	3.310167833
6	-1.987226952	-5.725969566	-5.405032635
6	2.530307243	3.504676694	0.222147438
6	0.359295911	-1.546474913	5.983168175
6	-0.176314588	-5.480081290	-1.470596795
6	-0.893118345	3.085417719	2.851662821
6	-1.167972543	-5.062286448	3.430568076
6	-2.964447657	-6.783125902	-4.895195190
6	1.937290645	4.784898670	-0.360122295
6	-1.526173730	-3.141838608	5.626554844
6	-1.880524747	-4.706029059	-3.122211927
6	1.402853646	3.966787142	2.416431906
6	-0.569467539	-4.330167114	5.739110010

6	-2.867260375	-5.762989458	-2.622849052
6	0.822798976	5.253698938	1.823005132
6	-0.236101097	-2.283394439	3.676965343
6	-0.274266695	-6.500802011	-3.743042317
6	0.223411420	2.627599548	0.671081167
6	0.720323297	-3.472884905	3.792088098
6	-1.261597408	-7.558317158	-3.244623813
6	-0.357073545	3.917116995	0.083462806
6	0.816492234	-3.891186047	5.260647716
6	-2.080089924	-6.965026700	-2.095468591
6	-0.542872472	4.935903991	1.209879616
6	-1.621620484	-2.721920631	4.158188439
6	-1.060314188	-5.298341958	-4.269476536
6	1.598919158	2.947092303	1.295549344
1	0.721053337	-0.709627460	6.590629985
1	0.487797022	-5.169403049	-0.655243278
1	-1.554602235	2.682039323	3.625033155
1	-1.531457033	-5.897543583	2.821922810
1	-3.628099239	-7.092531323	-5.709878561
1	2.610028261	5.182700817	-1.124675502
6	-5.183066921	0.806260979	2.088328924
6	-5.766555151	1.225261046	0.745683280
6	-4.840492889	0.780379219	-0.377891089
1	-5.838576846	1.120512727	2.905186363
1	-6.747728606	0.767245636	0.611599058
1	-5.905884839	2.308838007	0.712330294
1	-5.254728500	1.080995337	-1.344920603
6	-4.973511212	-0.701017472	2.134874755
6	-3.808567170	1.504203903	2.265401200
6	-3.453807005	1.480632601	-0.221213521
6	-4.624069275	-0.731619351	-0.351582013
6	-4.042686514	-1.120429754	1.005467699
1	-4.555432171	-0.992953583	3.100600903
1	-5.932314277	-1.210987878	2.026144775
6	-2.986901196	1.033989979	1.119159428
1	-3.956833772	2.584216979	2.259705631
1	-3.380566016	1.208182044	3.223993667
6	-2.539619739	1.038435633	-1.362365432
1	-3.615076404	2.562731926	-0.251980832
6	-3.688081286	-1.166325713	-1.477656007
1	-5.589903882	-1.231186312	-0.480209675
6	-2.660083093	-0.416801295	1.165337713
1	-3.872343513	-2.200303716	1.044929283
6	-2.338535683	-0.473638713	-1.308998017
1	-2.996326040	1.319939886	-2.312143503
1	-1.578427659	1.549621148	-1.297269261
1	-3.560648626	-2.250015589	-1.456488628
1	-4.121664380	-0.904484154	-2.443364106
6	-1.730385837	-0.855712274	0.037410681
1	-2.242625552	-0.697299963	2.136086298
1	-1.668115667	-0.780999940	-2.115545371
1	-0.751978111	-0.387318061	0.157282080
1	-1.584325846	-1.935684621	0.089580025
1	-5.310538245	4.017610428	-4.897881293
6	-5.557974821	4.708067728	-4.082328422

1	-5.750292597	0.299762713	5.673580727
6	-6.149920162	-0.564810274	6.221883483
1	-5.608455527	6.433284731	-5.371749151
6	-5.094711268	6.117769841	-4.457073039
1	-6.779103794	-1.594886947	4.432135733
6	-7.224580982	-1.249930533	5.374287872
6	-5.187041488	5.225660369	-1.675365603
1	-4.672022302	4.911753769	-0.755652191
1	-7.771434146	-0.946546054	9.234894741
6	-7.325480249	-1.287974633	8.294002252
1	-4.966800477	7.325465935	-1.234437113
6	-4.722418356	6.634898159	-2.050793922
1	-8.801807220	-2.830618465	7.994731122
6	-8.400653638	-1.970990076	7.445420191
1	-5.142008407	8.088954425	-3.584727365
6	-5.458885944	7.074312146	-3.318689574
1	-8.521568055	-2.963056313	5.533807487
6	-7.761826162	-2.459435264	6.143285286
1	-5.144378542	3.253707582	-2.545987328
6	-4.822903492	4.269974676	-2.813265018
1	-6.024869306	0.420832482	8.135651134
6	-6.786581318	-0.078978503	7.526361344
6	-3.315253034	4.282155285	-3.052082673
1	-3.063240384	3.590344399	-3.859127696
1	-7.523213418	1.770936149	6.691336715
6	-7.917010332	0.902289807	7.225040453
1	-7.487110774	7.393221095	-3.983793848
6	-6.968028385	7.063024560	-3.081636565
1	-6.186668960	-3.800635702	5.516095198
6	-6.629464236	-3.438860124	6.447539756
1	-2.787105964	3.956007094	-2.151325552
1	-8.358631800	1.255840756	8.159117266
1	-7.220866224	7.758544898	-2.277319088
1	-7.023746800	-4.303071361	6.986441394
1	-3.264573464	7.136581214	-4.977747577
6	-3.586145018	6.131729238	-4.696558658
1	-9.117757090	-0.766257072	4.458840983
6	-8.358213323	-0.272824239	5.070176408
1	-7.394773429	3.682158013	-3.597590415
6	-7.066890632	4.693829439	-3.848308279
1	-4.243812225	-1.045354978	7.114107880
6	-5.015950223	-1.542460466	6.521693257
1	-2.886262613	7.659231965	-2.534512371
6	-3.212695488	6.647815264	-2.284553592
1	-10.304381644	-1.487709038	6.551077664
6	-9.533547769	-0.991486452	7.144328551
1	-7.021656571	4.208191975	-1.151136257
6	-6.695275073	5.211857140	-1.438844717
1	-5.434037450	-1.769212579	9.216058295
6	-6.189750002	-2.262298182	8.599638010
1	-2.693122668	6.344635877	-1.370409963
1	-9.993041553	-0.657384304	8.076945634
1	-6.946702677	5.891697923	-0.621955385
1	-6.573946231	-3.116151290	9.161647974
1	-3.333417232	5.457087340	-5.518207849

1	-7.974735122	0.582204720	4.504696990
1	-7.585215107	4.995849805	-4.760620208
1	-4.557975535	-1.881318654	5.587335643
6	-7.415739083	5.650826713	-2.711347620
6	-5.565095640	-2.741482966	7.291537223
6	-2.865415568	5.692338652	-3.424407211
6	-8.981312322	0.208115060	6.378527808
1	-8.497394546	5.638093240	-2.540558879
1	-4.750266600	-3.441770907	7.508827887
1	-1.782564743	5.695730846	-3.594173225
1	-9.792750999	0.910447271	6.159778252
1	-8.755558603	-0.972744229	-4.237967592
6	-7.900739496	-1.322752127	-4.828026031
1	-8.884061215	-4.877877013	1.870027494
6	-8.173044874	-5.204123532	1.102564687
1	-4.427819842	4.759761204	4.360988213
6	-5.496483574	4.983174882	4.234063452
1	-6.483656120	-0.908182061	-3.259416792
6	-6.628573996	-0.643181408	-4.316301426
1	-8.649167497	-7.289028377	1.359176063
6	-7.759602999	-6.649680862	1.385251962
1	-5.519576349	4.379673003	2.159002454
6	-5.780573765	5.265554502	2.756839893
1	-7.069219733	-1.184248661	-8.165373628
6	-6.920034509	-1.443412013	-7.111143567
1	-5.055791975	-4.125745753	0.106283539
6	-5.948804755	-4.766419845	0.077885223
1	-7.609225118	7.400144691	5.474848075
6	-7.345207962	6.517429242	4.882169137
1	-4.792513995	-1.107622306	-7.191082716
6	-5.649351861	-0.760656652	-6.600486452
1	-4.824420698	-6.542501221	-0.406187866
6	-5.536339246	-6.213205384	0.358981012
1	-8.696596709	7.013794984	3.280996082
6	-7.630412938	6.797250261	3.405377748
1	-4.516077708	-0.669177847	-4.772314135
6	-5.437211866	-1.146022273	-5.134659912
1	-6.489417194	-8.141207725	0.484150894
6	-6.780641451	-7.101082433	0.298718318
1	-7.487232510	5.743667638	1.531890702
6	-7.273775080	5.553069031	2.589685809
1	-9.028691166	-1.412579470	-6.661519445
6	-8.111724858	-0.938604993	-6.293838937
1	-7.225231276	-3.273072883	0.971185842
6	-6.929446503	-4.313996785	1.161520241
1	-5.637919292	6.040678006	6.107965165
6	-5.851491369	6.229302894	5.049290873
1	-9.102361091	0.927081053	-5.850084252
6	-8.241031018	0.577577052	-6.424850619
1	-6.988777444	-4.082113094	3.303055852
6	-6.280104132	-4.413250178	2.539832385
1	-3.961890922	7.219462165	4.709843352
6	-5.027389146	7.423847334	4.573138118
1	-5.147541227	-2.925944658	-3.947501795
6	-5.310379889	-2.661474656	-4.996481818

1	-8.306439936	-7.646438559	-1.127445958
6	-7.429828007	-6.997126586	-1.080293862
1	-7.853939280	3.473776283	2.464175754
6	-8.098226526	4.357898632	3.061087814
1	-8.405799173	0.847322552	-7.470001748
1	-5.402554481	-3.761113082	2.588406717
1	-5.276131223	8.304475815	5.168435404
1	-4.449918922	-3.015021394	-5.570681315
1	-6.725924753	-7.329048747	-1.847296763
1	-9.160825860	4.566245965	2.923728061
1	-5.843159417	1.356050254	-4.073661033
6	-6.751677712	0.873014258	-4.447392741
1	-6.824634569	-7.787666795	2.963640456
6	-7.105854284	-6.751810993	2.762124641
1	-5.155278291	6.643219740	1.218469607
6	-4.956659958	6.458660244	2.276895281
1	-8.693338199	-3.316201172	-5.050632555
6	-7.778515396	-2.838806087	-4.693132584
1	-9.150820943	-4.077484920	-0.458448414
6	-8.832930981	-5.105521733	-0.270829329
1	-6.106746192	3.606410585	5.773514688
6	-6.322083557	3.793482201	4.719081653
1	-4.858524484	1.238295874	-6.381920816
6	-5.774343754	0.756166306	-6.733752530
1	-4.573102168	-7.344039885	1.923643720
6	-4.878747413	-6.313074412	1.733724740
6	-6.810060580	7.992312114	2.924775139
1	-7.027149905	8.193449558	1.872421888
1	-7.694688056	-3.442749309	-7.357938914
6	-6.791857145	-2.960107075	-6.977991517
1	-6.889747512	-3.614612551	-1.481196314
6	-6.605210856	-4.655118157	-1.295775048
1	-7.982336140	5.137329547	6.417059109
6	-8.173880653	5.325803634	5.358042531
1	-5.910387411	1.025151319	-7.783242970
1	-3.981439309	-5.689070405	1.762461823
1	-7.079444383	8.881875297	3.498040756
1	-5.947438305	-3.314939686	-7.573812758
1	-5.900525722	-4.954865608	-2.074060106
1	-9.237149365	5.549248896	5.244725821
1	-7.593992951	1.229210910	-3.848110716
1	-7.813540310	-6.440063098	3.533383890
1	-3.889525819	6.246188805	2.385053569
1	-7.647933983	-3.111418682	-3.643259456
1	-9.722294835	-5.738474015	-0.297951386
1	-6.047733071	2.894961702	4.158347719
6	-6.584877890	-3.328126994	-5.510281375
6	-7.841723787	-5.550417685	-1.343653203
6	-7.808939448	4.091414199	4.536596109
6	-6.966025749	1.243583060	-5.913077642
6	-5.867646809	-5.859780199	2.805782722
6	-5.323004538	7.692497755	3.098827238
1	-6.495210824	-4.415991310	-5.412889993
1	-8.308962024	-5.475327065	-2.331874863
1	-8.401600601	3.234086715	4.876406700

1	-7.057103331	2.331529277	-6.005787629
1	-5.398031962	-5.932584072	3.793845482
1	-4.732053071	8.548635260	2.755569874
6	-10.141381496	-1.348823279	0.534179408
6	-9.011843879	-0.976056708	-0.422651789
6	-9.399864560	0.273722619	-1.209083413
1	-9.857487115	-2.238734992	1.108564129
1	-8.091598506	-0.797795417	0.144232601
1	-8.819750045	-1.798509439	-1.117242249
1	-8.580205703	0.540000866	-1.889010595
6	-10.401833760	-0.187426242	1.490701903
6	-11.405266659	-1.637201480	-0.271907954
6	-10.665668069	-0.001607691	-2.025256787
6	-9.672501169	1.445674831	-0.263121012
6	-10.799134182	1.053504891	0.694645256
1	-11.203104831	-0.453238517	2.183115850
1	-9.504715418	0.022296076	2.080996888
6	-11.794026964	-0.393053848	-1.068023921
1	-11.232096097	-2.475435502	-0.951335380
1	-12.218557287	-1.917000377	0.400390942
6	-11.057578124	1.239023039	-2.824889414
1	-10.476807064	-0.834146967	-2.714202331
6	-10.062128141	2.687675659	-1.061181751
1	-8.768881976	1.666074919	0.320723297
6	-12.064617266	0.777278561	-0.119903958
1	-10.985547866	1.881265751	1.388445815
1	-12.699261468	-0.604721233	-1.647764161
6	-11.326133290	2.398017629	-1.867180528
1	-10.253809975	1.506708708	-3.516565292
1	-11.950619644	1.029800771	-3.417381643
1	-10.235040704	3.524205766	-0.380739967
1	-9.248275411	2.969870085	-1.734810990
6	-12.459221584	2.019629256	-0.915726970
1	-12.879713463	0.506522955	0.560868296
1	-11.608996548	3.288529368	-2.438931538
1	-13.370694035	1.821611295	-1.483619760
1	-12.664029498	2.848866670	-0.234824262
6	4.506586207	-4.991317616	-8.165582555
1	5.302909482	-4.433322146	-8.673170231
6	5.094800423	-6.270902239	-7.567286713
1	5.877896742	-6.005537595	-6.846520614
6	2.316208411	-6.134597995	-8.459672267
1	1.532850865	-6.399566339	-9.180008297
1	2.108572220	-7.973280255	-7.351179261
6	2.903496811	-7.414198476	-7.860399930
1	4.398104759	-7.944471331	-6.399600793
6	3.981903547	-7.032538248	-6.843790868
1	3.009887945	-4.459715057	-9.624089808
6	3.428367879	-5.371980895	-9.182716803
6	4.029169969	-6.237428598	-10.288003827
1	4.812364458	-5.681438135	-10.809162125
6	3.383980520	-6.162849840	-5.739747576
1	4.160020167	-5.903284209	-5.015345465
1	3.258160503	-6.493315995	-11.018710921
1	2.604528784	-6.716836430	-5.211131090

1	6.127939372	-8.043980310	-8.235269722
6	5.697560084	-7.138826599	-8.670066741
6	3.907529769	-4.120203162	-7.063584210
1	3.503654046	-3.203069147	-7.499329392
6	3.503218215	-8.283675711	-8.963797581
1	3.908579920	-9.201993853	-8.532521823
1	1.286364462	-4.358188612	-7.797285495
6	1.714337286	-5.263755702	-7.359326066
1	2.725404441	-8.564190656	-9.678104132
1	0.910131412	-5.805367258	-6.855524824
1	6.500559558	-6.594261333	-9.172528004
1	4.686184615	-3.839870549	-6.349533644
6	2.799128404	-4.892018962	-6.351613446
6	4.610821023	-7.510421942	-9.676703663
1	2.364661822	-4.269024255	-5.560793987
1	5.041485619	-8.133562151	-10.468029074
6	4.577391773	0.703186738	-12.313607494
1	5.566618033	0.488027571	-12.734332319
6	4.685491266	0.787491223	-10.789653389
1	5.381754242	1.591907343	-10.522843750
6	2.235368720	-0.110000034	-12.103568546
1	1.536631144	-0.911752746	-12.371862769
1	1.351148152	0.192114111	-10.163762150
6	2.342952452	-0.025295221	-10.579905160
1	3.381937838	1.180486988	-9.121562811
6	3.304813895	1.107156116	-10.213780695
1	3.544763250	-0.506768409	-13.769703942
6	3.616625253	-0.430896937	-12.678602113
6	4.126835753	-1.756117592	-12.114114726
1	5.106631631	-1.986579319	-12.538549047
6	2.793789999	2.433023804	-10.772595609
1	3.477225020	3.239199998	-10.495220533
1	3.444731861	-2.564567251	-12.388776518
1	1.814002476	2.662064185	-10.345215799
1	5.282761917	-0.464445246	-9.133371977
6	5.197157815	-0.535067143	-10.221173133
6	4.067930876	2.028914479	-12.877075493
1	4.005280560	1.968613298	-13.965936705
6	2.851856587	-1.348855260	-10.012610500
1	2.916131372	-1.284205469	-8.922201374
1	1.633566330	1.145524004	-13.752551840
6	1.722361449	1.214584291	-12.666123301
1	2.152893449	-2.152018334	-10.262315212
1	0.729595728	1.428913241	-12.262008090
1	6.189992953	-0.751539015	-10.622023946
1	4.766182901	2.830872289	-12.625621937
6	2.690490144	2.335470468	-12.293193066
6	4.229685321	-1.655909356	-10.594218769
1	2.324245934	3.285923229	-12.697310554
1	4.596024180	-2.605158999	-10.188246835
6	4.097996815	3.117380164	-4.926312677
1	3.124777906	2.797441179	-5.317462214
6	4.717959533	4.128586301	-5.891851626
1	4.052534916	4.996136504	-5.982346078
6	6.367331348	2.355097118	-4.254604697

1	7.030175671	1.486753375	-4.161288548
1	7.963064097	3.685907306	-4.829527044
6	6.990070460	3.366305804	-5.220362221
1	6.512674014	5.317820626	-6.004076577
6	6.065573159	4.582209728	-5.325811783
1	4.569647364	1.169263942	-4.137361274
6	5.019526442	1.901348322	-4.819810638
6	5.209485989	1.262812231	-6.194108294
1	4.244409473	0.927185494	-6.584449523
6	5.869838355	5.219803870	-3.951221056
1	5.218769704	6.093489998	-4.037140753
1	5.858489863	0.387230040	-6.110168044
1	6.831753914	5.556703937	-3.558517716
1	5.340190767	4.222425146	-7.955355169
6	4.907729439	3.492519274	-7.267450762
6	3.899360536	3.748035668	-3.550553360
1	3.444561285	3.018076948	-2.874287228
6	7.183401936	2.730268460	-6.595572202
1	7.640787412	3.451821008	-7.276156857
1	5.752187187	2.261589672	-2.186213762
6	6.177347129	2.992276014	-2.879445399
1	7.855701144	1.872680795	-6.516168798
1	7.144645564	3.305176123	-2.480308961
1	3.939868690	3.185716978	-7.672490241
1	3.221125210	4.602273483	-3.628734459
6	5.249779283	4.199557691	-2.998786744
6	5.831199881	2.282291934	-7.145945606
1	5.110752044	4.654956915	-2.010605678
1	5.970127383	1.826179044	-8.131647083
6	-11.665241898	-5.697913163	3.326252264
1	-11.147540431	-6.464425211	2.736456378
6	-10.736737417	-5.208926713	4.440248969
1	-9.829891353	-4.778247795	3.994105093
6	-13.639836148	-5.225282429	4.767753631
1	-14.544687561	-5.652517600	5.214800464
1	-13.225252251	-3.965830977	6.469258607
6	-12.710846513	-4.735247648	5.880814863
1	-10.795025233	-3.766551866	6.040358863
6	-11.459247459	-4.127283306	5.245126528
1	-13.580687709	-6.674464249	3.171700037
6	-12.916109145	-6.308415937	3.963196851
6	-12.529791268	-7.471469031	4.874944134
1	-12.027386083	-8.248036897	4.292918016
6	-11.842781132	-2.963408548	4.334047968
1	-10.943321541	-2.523312313	3.891852764
1	-13.427843874	-7.911162163	5.314373440
1	-12.346842458	-2.189161937	4.917477135
1	-9.668444713	-6.016914499	6.132756704
6	-10.346389136	-6.370566697	5.351166280
6	-12.055669881	-4.537469298	2.412768955
1	-12.711278350	-4.897679832	1.616620849
6	-12.322994064	-5.895310042	6.795266511
1	-11.671421270	-5.534894553	7.595385959
1	-14.704721622	-4.411265232	3.075051821
6	-14.028592617	-4.061150991	3.858446390

1	-13.217893704	-6.317635604	7.257422018
1	-14.553695888	-3.296752337	4.436492175
1	-9.822697212	-7.136601179	4.773430111
1	-11.164436605	-4.115046460	1.941951897
6	-12.771442054	-3.464953695	3.230524806
6	-11.603426836	-6.967743300	5.979936879
1	-13.051178284	-2.629381598	2.577865933
1	-11.324910131	-7.801268511	6.634070147
6	-10.413705118	8.017156104	0.884593069
1	-9.497536579	8.322132738	1.404377886
6	-10.996976630	9.227216750	0.151898098
1	-10.263580436	9.595748976	-0.575985187
6	-12.703397543	7.087642099	1.172279758
1	-13.436709152	6.721197888	1.899662622
1	-14.203916322	7.996570937	-0.079694006
6	-13.286552666	8.297668676	0.439805791
1	-12.681022393	9.651191128	-1.125849391
6	-12.264922068	8.792476717	-0.586667402
1	-11.019279429	6.656114846	2.445745731
6	-11.434696440	7.520285435	1.910355539
6	-11.757171150	8.628155261	2.910231408
1	-10.849540388	8.923543167	3.443557039
6	-11.939123729	7.681517533	-1.583472138
1	-11.219621737	8.044226505	-2.321957967
1	-12.475907536	8.264739492	3.649147065
1	-12.845276508	7.384368122	-2.116463987
1	-11.723201542	11.204406279	0.620758384
6	-11.319746320	10.337921818	1.149344459
6	-10.084830340	6.906958457	-0.110479077
1	-9.653943480	6.050482507	0.415740920
6	-13.613066700	9.406372693	1.438375653
1	-14.042635564	10.262747997	0.913826844
1	-11.969188318	5.111962924	0.715010549
6	-12.376654337	5.974473982	0.179363108
1	-14.353008138	9.049359416	2.158740083
1	-13.286561335	5.652588970	-0.332905405
1	-10.406946463	10.653062029	1.661409160
1	-9.346736538	7.265039754	-0.832282095
6	-11.357952881	6.482039350	-0.838671337
6	-12.338790683	9.828888507	2.166849029
1	-11.123497123	5.684378526	-1.553555084
1	-12.571797987	10.624841087	2.882526120
6	-12.350916572	2.287818731	4.292565230
1	-11.525938755	1.617612613	4.017699740
6	-12.655117749	2.127289844	5.783884216
1	-11.761618387	2.388185745	6.365627525
6	-14.736654691	2.858786567	3.869969379
1	-15.629711560	2.600431164	3.288831099
1	-15.864875999	3.363642503	5.636698722
6	-15.040492886	2.695876277	5.361035222
1	-14.006349599	2.976476473	7.232551070
6	-13.793284723	3.077624975	6.161906399
1	-13.385514561	2.012979467	2.420837646
6	-13.598542478	1.908574105	3.491513527
6	-13.995774215	0.464788550	3.789557545

1	-13.183710166	-0.209386475	3.505171022
6	-13.394303102	4.521206858	5.861859722
1	-12.512270611	4.791900412	6.448193973
1	-14.877402928	0.192934173	3.204071294
1	-14.204777444	5.196214840	6.145683198
1	-13.249159467	0.568032929	7.153169903
6	-13.049958514	0.683452549	6.085251550
6	-11.950896757	3.730158171	3.991521317
1	-11.721940207	3.837375079	2.927116385
6	-15.439350528	1.252412226	5.662347248
1	-15.668048556	1.145188525	6.725240245
1	-14.135450712	4.409054360	2.496106289
6	-14.338011621	4.301196049	3.564873203
1	-16.337937960	0.990855267	5.098659828
1	-15.159139908	4.974834539	3.820616202
1	-12.225026306	0.015545813	5.824857282
1	-11.052294328	3.988297974	4.557991744
6	-13.094230664	4.667498796	4.371783042
6	-14.294726985	0.316873538	5.279769481
1	-12.805746676	5.702562438	4.156131712
1	-14.580766541	-0.719165146	5.495944496

Table S5. Geometries of diamantane (**D**), 1-diamantyl cation (**Dp**) and cluster **CL2** in Cartesian coordinates in Å computed at the B3LYP-D3(BJ)/def2-TZVPP level of theory at 0.4 K.

**D**			
1	-6.919405000	-0.684186000	4.112531000
6	-6.672461000	-0.929893000	5.150127000
1	-6.649210000	-3.060076000	4.789943000
6	-7.160817000	-2.360836000	5.457925000
1	-7.545116000	0.408312000	8.210649000
6	-7.033480000	-0.290910000	7.542642000
1	-7.274893000	-1.967487000	8.888117000
6	-7.521811000	-1.721773000	7.850513000
1	-7.120843000	-3.721523000	7.137945000
6	-6.788402000	-2.702670000	6.915427000
1	-7.073275000	1.069765000	5.862804000
6	-7.405775000	0.050895000	6.085162000
1	-9.182921000	0.223808000	4.859005000
6	-8.920786000	-0.049895000	5.885330000
1	-4.764508000	-3.308742000	6.454409000
6	-5.273450000	-2.601713000	7.115411000
1	-9.429453000	0.657097000	6.546556000
1	-5.011430000	-2.875362000	8.141775000
1	-9.011449000	-3.483198000	5.458955000
6	-8.675658000	-2.462211000	5.255161000
1	-4.809216000	0.173792000	5.105482000
6	-5.157656000	-0.833076000	5.353750000
1	-9.385017000	-2.825508000	7.895116000
6	-9.036572000	-1.818639000	7.646854000
1	-5.182849000	0.831509000	7.541898000
6	-5.518700000	-0.189522000	7.745506000
1	-9.545687000	-1.126426000	8.323200000
1	-5.263433000	-0.411230000	8.785941000
1	-8.931043000	-2.240631000	4.214730000

1	-4.648547000	-1.525322000	4.677467000
6	-4.804717000	-1.171873000	6.808941000
6	-9.389542000	-1.479740000	6.191702000
1	-3.724204000	-1.097992000	6.954890000
1	-10.470087000	-1.553640000	6.046003000

**Dp**			
1	4.616992000	-0.805307000	-3.878954000
1	6.457157000	-0.880542000	0.023944000
1	3.600035000	-3.008865000	-3.391293000
1	6.311593000	0.151226000	-2.276359000
1	5.406887000	-3.089614000	0.425784000
1	3.038739000	-2.226588000	0.648351000
1	2.360740000	-0.903762000	-2.806440000
1	4.729754000	0.907218000	-0.535416000
1	5.871620000	-2.856135000	-4.507830000
1	8.112632000	-1.651888000	-1.719150000
1	6.033747000	-4.732299000	-1.245392000
1	4.197527000	-0.991054000	1.091856000
1	1.940510000	-2.174939000	-1.676817000
1	3.971995000	0.943105000	-2.115749000
1	7.006583000	-1.601684000	-4.064194000
1	7.702601000	-2.937051000	-0.606422000
1	5.265316000	-4.704433000	-2.864122000
6	5.632767000	-4.103468000	-2.036314000
6	6.225303000	-2.254413000	-3.670056000
6	7.297569000	-2.301151000	-1.394577000
6	2.754068000	-1.524890000	-2.001274000
6	4.368277000	0.274752000	-1.348819000
6	3.819101000	-1.574828000	0.252474000
6	6.808920000	-3.139841000	-2.573007000
6	3.263761000	-0.661321000	-0.843679000
6	6.154209000	-1.448077000	-0.856460000
6	4.686232000	-3.100977000	-1.570016000
6	5.093424000	-1.404979000	-3.103294000
6	5.538953000	-0.536804000	-1.922879000
6	4.960525000	-2.425552000	-0.311676000
6	3.898811000	-2.379562000	-2.555342000
1	7.587780000	-3.798369000	-2.953981000
1	2.440484000	-0.071582000	-0.440365000

**CL2**			
1	-0.996108000	0.145457000	-1.994674000
1	0.776530000	1.889518000	-2.001402000
1	2.004285000	-2.875165000	-1.418604000
1	3.768082000	-1.125488000	-1.410082000
1	3.158916000	1.214310000	-2.001774000
1	-0.380542000	-2.204357000	-1.424296000
1	-0.520726000	-1.591488000	-3.846303000
1	2.111276000	1.828198000	0.171535000
1	0.704863000	-2.818351000	-3.579555000
1	3.333072000	0.587019000	0.400407000
1	1.847248000	1.286219000	-4.161328000
1	-0.832300000	-0.481406000	0.407016000
1	3.627962000	-0.492257000	-3.810973000

1	0.928353000	-2.270359000	0.743377000
1	3.163440000	-2.170295000	-3.581411000
1	2.632175000	-1.847769000	0.754287000
1	0.149617000	0.847005000	-4.182008000
1	-0.344449000	1.194970000	0.164065000
6	-0.066793000	0.157779000	-0.043029000
6	1.113175000	0.611646000	-3.716615000
6	1.670929000	-1.604040000	0.296343000
6	2.866090000	-1.139857000	-3.370952000
6	2.353936000	0.783625000	-0.042154000
6	0.449945000	-1.774947000	-3.380195000
6	1.303979000	-0.142722000	0.581882000
6	1.499253000	-0.846949000	-4.000152000
6	1.739427000	-1.833989000	-1.215800000
6	2.794381000	-0.911278000	-1.858413000
6	0.001119000	-0.079240000	-1.553133000
6	1.042435000	0.847140000	-2.204668000
6	2.414329000	0.551163000	-1.554218000
6	0.368719000	-1.540355000	-1.867186000
1	1.254993000	0.027062000	1.658990000
1	1.545004000	-1.014892000	-5.077956000
6	-4.690910000	-0.913296000	-3.807075000
6	-5.615556000	-0.717173000	-2.609384000
6	-5.252860000	0.575511000	-1.885009000
1	-4.895717000	-1.849796000	-4.324507000
1	-6.651929000	-0.669036000	-2.949440000
1	-5.549458000	-1.565653000	-1.926480000
1	-5.859332000	0.716619000	-0.989975000
6	-4.769022000	0.282171000	-4.755887000
6	-3.189836000	-1.010948000	-3.266891000
6	-3.725902000	0.480859000	-1.333176000
6	-5.329750000	1.805848000	-2.799052000
6	-4.418085000	1.564670000	-4.007304000
1	-4.097861000	0.140998000	-5.604393000
1	-5.778619000	0.363326000	-5.163228000
6	-3.047093000	0.287444000	-2.611884000
1	-3.103433000	-1.850896000	-2.581998000
1	-2.523231000	-1.140087000	-4.113433000
6	-3.383706000	1.773453000	-0.588793000
1	-3.662414000	-0.402351000	-0.700144000
6	-4.935538000	3.085389000	-2.049710000
1	-6.356622000	1.903758000	-3.160069000
6	-2.894823000	1.469562000	-3.455075000
1	-4.418179000	2.425718000	-4.676928000
6	-3.495316000	2.970896000	-1.537158000
1	-4.069641000	1.886333000	0.251628000
1	-2.379803000	1.695738000	-0.174683000
1	-5.040464000	3.945215000	-2.714455000
1	-5.618129000	3.246240000	-1.213466000
6	-2.540505000	2.763478000	-2.716394000
1	-2.252852000	1.277872000	-4.312206000
1	-3.221156000	3.881485000	-1.003592000
1	-1.505718000	2.715159000	-2.375076000
1	-2.607028000	3.594260000	-3.419838000

Table S6. Geometries of diamantane (**D**), 1-diamantyl cation (**Dp**) and cluster **CL2** in Cartesian coordinates in Å computed at the B3LYP/def2-TZVPP level of theory at 0.4 K.

**D**			
1	-6.914927000	-0.682410000	4.108622000
6	-6.670493000	-0.929030000	5.147510000
1	-6.652446000	-3.063477000	4.785970000
6	-7.162046000	-2.363009000	5.455612000
1	-7.541825000	0.411779000	8.214712000
6	-7.032220000	-0.288692000	7.545079000
1	-7.279316000	-1.969308000	8.892068000
6	-7.523765000	-1.722669000	7.853188000
1	-7.117076000	-3.725771000	7.139546000
6	-6.786774000	-2.705436000	6.916437000
1	-7.077180000	1.074071000	5.861158000
6	-7.407482000	0.053730000	6.084246000
1	-9.188732000	0.227013000	4.857421000
6	-8.925752000	-0.047948000	5.883931000
1	-4.758463000	-3.312096000	6.456912000
6	-5.268511000	-2.603748000	7.116759000
1	-9.435769000	0.660415000	6.543786000
1	-5.005535000	-2.878725000	8.143267000
1	-9.016769000	-3.485942000	5.455361000
6	-8.680215000	-2.464161000	5.252928000
1	-4.802802000	0.174842000	5.102603000
6	-5.152466000	-0.832003000	5.352198000
1	-9.391463000	-2.826543000	7.898094000
6	-9.041791000	-1.819700000	7.648502000
1	-5.177498000	0.834252000	7.545347000
6	-5.514053000	-0.187530000	7.747771000
1	-9.552036000	-1.128502000	8.326162000
1	-5.257473000	-0.407413000	8.788994000
1	-8.936795000	-2.244283000	4.211703000
1	-4.642219000	-1.523206000	4.674548000
6	-4.798727000	-1.171606000	6.809716000
6	-9.395534000	-1.480086000	6.190984000
1	-3.717451000	-1.097679000	6.955692000
1	-10.476811000	-1.554025000	6.045039000

**Dp**			
1	4.620582000	-0.805329000	-3.884785000
1	6.463976000	-0.880474000	0.024761000
1	3.595979000	-3.009718000	-3.391661000
1	6.311143000	0.158016000	-2.276142000
1	5.404683000	-3.090495000	0.429107000
1	3.035280000	-2.227826000	0.651555000
1	2.352207000	-0.904554000	-2.805915000
1	4.726508000	0.913435000	-0.534454000
1	5.876372000	-2.857142000	-4.513345000
1	8.119956000	-1.654804000	-1.719759000
1	6.032229000	-4.736808000	-1.245319000
1	4.191507000	-0.992381000	1.098364000
1	1.935640000	-2.175862000	-1.676425000
1	3.969015000	0.949080000	-2.113676000
1	7.011638000	-1.604444000	-4.069507000

1	7.709822000	-2.938137000	-0.606515000
1	5.264167000	-4.708886000	-2.863185000
6	5.633794000	-4.106150000	-2.036886000
6	6.229386000	-2.256198000	-3.673843000
6	7.303084000	-2.302977000	-1.395331000
6	2.749078000	-1.524389000	-2.000620000
6	4.365531000	0.279014000	-1.347429000
6	3.815434000	-1.574502000	0.255951000
6	6.812546000	-3.143601000	-2.574762000
6	3.259102000	-0.659018000	-0.841350000
6	6.157963000	-1.447452000	-0.856004000
6	4.688893000	-3.098685000	-1.571196000
6	5.095399000	-1.404391000	-3.106456000
6	5.539809000	-0.533027000	-1.923272000
6	4.960215000	-2.425018000	-0.309278000
6	3.896646000	-2.379066000	-2.556490000
1	7.591755000	-3.802880000	-2.955881000
1	2.435272000	-0.069005000	-0.437722000

**CL2**			
1	-0.740306000	0.072392000	-1.856089000
1	1.020452000	1.823549000	-1.922110000
1	2.289267000	-2.945670000	-1.373349000
1	4.049298000	-1.190530000	-1.433828000
1	3.417055000	1.152830000	-1.993164000
1	-0.100863000	-2.280363000	-1.304651000
1	-0.341383000	-1.644220000	-3.713479000
1	2.443465000	1.763977000	0.216875000
1	0.887448000	-2.885557000	-3.510416000
1	3.668897000	0.519727000	0.403359000
1	2.038636000	1.220461000	-4.111111000
1	-0.491181000	-0.572621000	0.545399000
1	3.822889000	-0.565865000	-3.832903000
1	1.282136000	-2.342704000	0.822501000
1	3.366298000	-2.242345000	-3.581127000
1	2.983616000	-1.914120000	0.777742000
1	0.339551000	0.785798000	-4.087466000
1	-0.048391000	1.113093000	0.298322000
6	0.248014000	0.082828000	0.073417000
6	1.314778000	0.547051000	-3.645654000
6	2.006683000	-1.672268000	0.350918000
6	3.075717000	-1.210637000	-3.362707000
6	2.675277000	0.718302000	-0.007088000
6	0.650951000	-1.840941000	-3.290116000
6	1.643198000	-0.208968000	0.651148000
6	1.685186000	-0.914783000	-3.945840000
6	2.025422000	-1.903864000	-1.166422000
6	3.058358000	-0.977580000	-1.845892000
6	0.261551000	-0.147148000	-1.444912000
6	1.289701000	0.781097000	-2.127811000
6	2.685147000	0.488486000	-1.524691000
6	0.631185000	-1.612356000	-1.771265000
1	1.633987000	-0.042001000	1.730757000
1	1.694869000	-1.081583000	-5.025844000
6	-4.954392000	-0.838788000	-3.897318000

6	-5.906346000	-0.640272000	-2.718271000
6	-5.558093000	0.652208000	-1.980359000
1	-5.148618000	-1.774476000	-4.421297000
1	-6.935331000	-0.588995000	-3.081662000
1	-5.861182000	-1.490929000	-2.035564000
1	-6.181898000	0.789485000	-1.095930000
6	-5.002234000	0.361737000	-4.843243000
6	-3.461866000	-0.948269000	-3.307587000
6	-4.032955000	0.545130000	-1.383699000
6	-5.598637000	1.891083000	-2.887547000
6	-4.665392000	1.641710000	-4.080429000
1	-4.311474000	0.222313000	-5.676975000
1	-6.000504000	0.447277000	-5.278836000
6	-3.352108000	0.352115000	-2.655278000
1	-3.415371000	-1.786954000	-2.616585000
1	-2.762158000	-1.090391000	-4.127171000
6	-3.685373000	1.833503000	-0.628444000
1	-4.000243000	-0.342752000	-0.754638000
6	-5.203937000	3.164221000	-2.118525000
1	-6.617159000	2.008063000	-3.269072000
6	-3.144790000	1.530813000	-3.485593000
1	-4.635118000	2.504559000	-4.747955000
6	-3.772189000	3.036402000	-1.576248000
1	-4.375541000	1.950933000	0.208461000
1	-2.683031000	1.742534000	-0.205921000
1	-5.289604000	4.030708000	-2.778225000
1	-5.903636000	3.326674000	-1.296084000
6	-2.797125000	2.826873000	-2.740860000
1	-2.488699000	1.327283000	-4.330085000
1	-3.502633000	3.944505000	-1.034995000
1	-1.763017000	2.779526000	-2.393990000
1	-2.854122000	3.657375000	-3.446628000
